# A continuum mechanical framework for modeling tumor growth and treatment in two- and three-phase systems

**DOI:** 10.1007/s00419-021-01891-8

**Published:** 2021-06-09

**Authors:** Cass T. Miller, William G. Gray, Bernhard A. Schrefler

**Affiliations:** Department of Environmental Sciences and Engineering, University of North Carolina, Chapel Hill, NC, USA; Department of Environmental Sciences and Engineering, University of North Carolina, Chapel Hill, NC, USA; Department of Civil, Environmental and Architectural Engineering, University of Padua, Padua, Italy

**Keywords:** TCAT, Porous medium, Multiphase flow, Species transport, Model formulation, Mass transfer, Reactions

## Abstract

The growth and treatment of tumors is an important problem to society that involves the manifestation of cellular phenomena at length scales on the order of centimeters. Continuum mechanical approaches are being increasingly used to model tumors at the largest length scales of concern. The issue of how to best connect such descriptions to smaller-scale descriptions remains open. We formulate a framework to derive macroscale models of tumor behavior using the thermodynamically constrained averaging theory (TCAT), which provides a firm connection with the microscale and constraints on permissible forms of closure relations. We build on developments in the porous medium mechanics literature to formulate fundamental entropy inequality expressions for a general class of three-phase, compositional models at the macroscale. We use the general framework derived to formulate two classes of models, a two-phase model and a three-phase model. The general TCAT framework derived forms the basis for a wide range of potential models of varying sophistication, which can be derived, approximated, and applied to understand not only tumor growth but also the effectiveness of various treatment modalities.

## 1 Introduction

Tumor growth and treatment is an area of science of significant interest to society. Ideally, scientists wish to understand fundamental aspects of tumor growth in sufficient detail to enable accurate mathematical models of the behavior at the length scale of interest in humans, which is on the order of centimeters. The problem that arises is a common problem in science and applied mathematics: how to most efficiently and effectively account for important small-scale behavior in larger scale models. To understand the issue more completely, one must consider the scales involved, which we will identify as the molecular scale, the microscale, and the macroscale.

At the molecular scale, one might endeavor, for example, to understand the processes and reactions that lead to the damage and repair of DNA, gene variations important for specific types of cancer, the role of environmental factors, and interactions among contributing factors. Within this context, important fundamental understanding, such as the hallmarks of cancer [24,25], emerges. Such fundamental, small-scale work is a principal focus of the medical research community and much has been learned over the last few decades. However, a disconnect exists between the molecular scale of such fundamental work and the typical length scale of tumors in humans. As a result, it is not obvious how molecular-scale studies can be used to describe tumor growth [39]. Because of this, tumor growth is often described based upon purely statistical representations of empirical fits to observations [7,8,27,34,58,62]. While such fits to data may be good, mechanistic understanding is lacking from such approaches. Put another way, empirical fits are not based on system physics and thus provide an insufficient basis fundamentally to describe factors affecting tumor growth and also to make meaningful, mechanistically based descriptions of how fundamental changes in a system will affect tumor growth.

As an alternative, microscale continuum methods can be used to describe tumor growth. The microscale is a small scale at which continuum mechanical approaches are valid. Even though tumors occur in biological systems, it can be reasoned that common continuum mechanical notions such as conservation principles, mass transfer, reactions, and thermodynamics are applicable and are relatively well understood at the microscale. The challenge that emerges from a microscale modeling approach is the need to represent meaningfully the processes that occur in a wide variety of matter types including healthy tissue, active tumor regions, necrotic tumor regions, extra-cellular matrix regions, and blood vessels. At the microscale, the domains of each of these entities change with time; interfaces form between the entities; and common curves form where three entities meet. Because of this inherent complexity, and the length scales of interest, microscale modeling approaches are not practical or feasible for the mechanistic description of the dynamics of tumors in a living being.

Just as averaging of molecular-scale phenomena is necessary to formulate a microscale continuum model that abstracts away a portion of the mechanistic detail, larger-scale averaging can be performed to derive a macroscale representation from a microscale formulation. At the macroscale, one endeavors to describe the dynamics of the entities (phases, interfaces, common curves) involved in an averaged sense with notions such as volume fractions and other specific entity measures—concepts that do not exist at the microscale. A point in a macroscale model thus represents the averaged conditions embodying all entities around the centroid of a small region. Such models can be formulated in a deterministic sense if and when the averaged conditions are insensitive to small changes in the scale of the averaging region. Useful macroscale models must account for subscale behavior in an approximate, averaged sense; but they must also mechanistically describe tumor dynamics at the scale of applications.

A variety of approaches exist to formulate macroscale models of tumor growth [57]. More broadly considered, a variety of homogenization and averaging methods have been applied to develop macroscale models of porous medium systems [2,21]. Typically macroscale model formulations are formed and closed phenomenologically directly at the macroscale. Examples for this are the multiparameter models based on mixture theory [26,37,50,51]. While an expedient approach, phenomenological macroscale models do not provide a firm connection with the microscale and cannot be assured to be consistent with the second law of thermodynamics.

Recent continuum mechanics work has resulted in the development of the thermodynamically constrained averaging theory (TCAT) [15,19,44]. The TCAT approach formally averages microscale quantities to the macroscale, including not only phases but also interfaces and common curves, incorporates thermodynamics in a scale-consistent manner, and results in entropy inequality expressions that can be used to guide the formulation of models. TCAT also includes evolution equations for the geometry of the phase regions and their boundaries that reduce the closure problem, are based upon mathematical theorems, and are separate from all conservation principles. Recently, notions from integral and differential geometry have been used rigorously to address closure relations needed to describe capillary pressure [42,47]. Because macroscale TCAT models are firmly connected with microscale antecedents, experimental observations or computational simulations at the microscale can be averaged to the macroscale and used to validate a resultant macroscale model. Considerable microscale resources exist, which can be leveraged to advance macroscale models.

While some aspects of TCAT formulations have been used to model tumor growth [31,54], a complete and rigorous hierarchy of models formulated and closed using TCAT procedures has not yet been accomplished. Such an advancement is possible by leveraging recent advances in the TCAT approach and applying these to tumor growth and treatment. This advancement provides the development of a hierarchy of models of varying sophistication that can be applied to this important class of problems.

## 2 Goal and objectives

The overall goal of this work is to develop a framework for modeling tumor growth and treatment at the macroscale. The specific objectives of this work are: (1) to summarize available macroscale conservation, potential, and thermodynamic equations applicable to modeling tumors; (2) to provide a general simplified entropy inequality (SEI), which can be used to guide model closure; (3) to formulate a rigorous two-phase macroscale model for tumor growth; (4) to formulate a rigorous three-phase macroscale model for tumor growth; and (5) to discuss ways in which the model-building framework can be used to formulate models to describe a wide variety of more complex and detailed systems than the ones provided explicitly herein.

## 3 TCAT approach

The approach to be taken to meet the goal and objectives of this work is to leverage existing TCAT model-building components [19,52] to formulate models for tumor growth. The advantage of this approach is one of relative simplicity and expediency: using the available formulated components, model building is relatively straightforward; and the substantial amount of effort and manipulations needed to derive an SEI are eliminated. The disadvantage of this approach is that it could be viewed as jumping into the middle of a carefully structured model formulation approach. To circumvent this potential misperception, a brief summary of the TCAT approach is provided to orient readers who are not yet familiar with TCAT. General guidance for the TCAT approach is available in the literature [15,19,44–46], and specific details of a two-fluid phase compositional model for a porous medium system have also been presented [52].

In general, the TCAT approach is initiated with a general, minimal description of the system to be modeled. This description includes the entities to be modeled, specification of the physical and chemical phenomena occurring within each entity, and the interactions among entities. For the case of concern herein, entities considered include three phases, three interfaces, and a common curve. Entropy, momentum, and energy are resolved at the entity level, and the chemical composition of the mass of each entity is resolved. Classical irreversible thermodynamics is used, and continuum methods are assumed to be valid and deterministic at the macroscale.

All conservation, balance, thermodynamic, and potential equations are formulated at the microscale and then systematically averaged to the macroscale. The macroscale balance of entropy is arranged to solve for the entropy density production rate, which is known to be a nonnegative quantity from the second law of thermodynamics. All macroscale conservation and potential equations are arranged such that the terms in the equation sum to zero. Each collection of terms is multiplied by a Lagrange multiplier and added to a system entropy balance. The Lagrange multipliers are solved for to eliminate material derivatives to the extent possible, yield a dimensionally consistent equation, and connect the processes that produce entropy to the rate of entropy production. Rearrangement of this augmented entropy inequality and reduction to a strict flux–force form, requiring approximations, is a key archival result of the TCAT approach. All of the manipulations leading up to this equation do not need to be repeated for each application that uses the SEI for a given class of models. Furthermore, macroscale conservation and evolution equations are also already available and can be used to formulate models. The chief remaining work when leveraging an extant model hierarchy is thus to use the SEI to formulate model closure relations and combine these equations with conservation and evolution equations to produce a well-posed model. Because of the general approach taken that includes minimal assumptions, a typical SEI supports the formulation of a hierarchy of models of varying sophistication, which results from applying secondary restrictions to the general SEI (e.g. entities of importance and their properties, specific forms of closure relations). This brief overview of the TCAT approach will be detailed in the sections that follow and used to produce example tumor growth models.

We will consider tumor growth models that can be idealized as containing two fluid phases and one solid phase. Compositional effects for mass will be important. An existing TCAT model hierarchy that meets these specifications has been derived [52] and will be relied upon as a foundation for the TCAT modeling approach that follows. Use of this hierarchy will simplify the model formulation process.

## 4 Macroscale equations

Macroscale equations relied upon in the TCAT approach to form an entropy inequality include conservation equations, a balance of entropy equation, thermodynamic equations, and potential equations. In this section, we summarize only the conservation equations, which are used to construct the target models of concern in this work. Details of the derivation of these equations are available in the literature [19,52]. The approach followed in this work leverages available results without the burden of reproducing these model components—greatly simplifying the model-building process.

We make use of the conservation equations for mass, momentum, and energy, which can be written in a common form for all species in all entities. The compositional, mass–conservation equation for species *i* in entity *α* is

(1) $$M_{**}^{\bar{\bar{i\alpha }}}=\frac{{\text{D}}^{\bar{\alpha }}\left(\epsilon^{\bar{\bar{\alpha }}}\rho^{\alpha }\omega^{i\;\bar{\alpha }}\right)}{\text{D}t}+\epsilon^{\bar{\bar{\alpha }}}\rho^{\alpha }\omega^{i\;\bar{\alpha }}I:d^{\bar{\bar{\alpha }}}+\nabla \cdot \left(\epsilon^{\bar{\bar{\alpha }}}\rho^{\alpha }\omega^{i\;\bar{\alpha }}u^{\bar{\bar{i\alpha }}}\right)\;-\epsilon^{\bar{\bar{\alpha }}}r^{i\alpha }-\underset{\kappa \in J_{\text{c}\alpha }}{\sum }\overset{i\kappa \to i\alpha }{M}=0\text{ for }i\in J_{s},\alpha \in J,$$

where $\epsilon^{\bar{\bar{\alpha }}}$ is the entity extent measure (volume fraction, specific interfacial area, specific common curve length), *ρ*^*α*^ is the mass density, $\omega^{i\;\bar{\alpha }}$ is the mass fraction, **I** is the identity tensor, $d^{\bar{\bar{\alpha }}}$ is the rate of strain tensor, $u^{\bar{\bar{i\alpha }}}$ is the deviation velocity, *r*^*iα*^ is the rate of mass production of species *i* resulting from all reactions in entity *α*, $\overset{i\kappa \to i\alpha }{M}$ represents the rate of mass transfer of species *i* from connected entity *κ* to entity *α*, $J_{s}$ is the index set of chemical species, $J_{\text{c}\alpha }$ is the index set of connected entities, and J is the index set of all entities in the system. Superscripted entity and species qualifiers denote macroscale quantities.

In general, the set $J_{\text{c}\alpha }$ may contain entities of lower dimensions and higher dimensions than the dimension of the *α* entity. For example, the closed set of the solid phase adjoins the wetting fluid phase and the non-wetting fluid phase at the interface that forms between the respective pairs of phases, and the common curve formed at the intersection of the solid phase and the two fluid phases. Thus, $J_{\text{cs}}=\left\{ws,ns,wns\right\}$, where *s* in an index specifying the solid phase, and the grouping of indices refer to the respective interface and common curve entities. We will restrict the inter-entity transfer of mass and entropy to entities of at most one dimension higher or lower than a reference entity. For momentum and energy, we will allow a concentrated force along the common curve to act on the solid phase in the most general case, and the inter-entity transfer of internal energy will include interactions between the common curve and the solid phase as well. These restrictions are incorporated into the form of the conservation and balance equations written.

Conservation of momentum may be considered from either a compositional or an overall entity perspective [17]. Taking the latter approach, the momentum equation is written as

(2) $$P_{**}^{\bar{\bar{\alpha }}}=\frac{{\text{D}}^{\bar{\alpha }}\left(\epsilon^{\bar{\bar{\alpha }}}\rho^{\alpha }v^{\bar{\alpha }}\right)}{\text{D}t}+\epsilon^{\bar{\bar{\alpha }}}\rho^{\alpha }v^{\bar{\alpha }}I:d^{\bar{\alpha }}-\underset{i\in J_{\text{c}}}{\sum }\epsilon^{\bar{\bar{\alpha }}}\rho^{\alpha }\omega^{i\;\bar{\alpha }}g^{\bar{i\alpha }}\;-\underset{\kappa \in J_{c\alpha }}{\sum }\underset{i\in J_{s}}{\sum }\overset{i\kappa \to i\alpha }{M}\left(v^{\bar{\bar{\alpha ,\kappa }}}+u_{i}^{\bar{\bar{\alpha ,\kappa }}}\right)-\underset{\kappa \in J_{c\alpha }}{\sum }\overset{\kappa \to \alpha }{T_{0}}\;-\nabla \cdot \left(\epsilon^{\bar{\bar{\alpha }}}t^{\bar{\bar{\alpha }}}\right)=0\text{ for }\alpha \in J,$$

where $v^{\bar{\alpha }}$ is the velocity, $g^{\bar{i\alpha }}$ is the body force per unit mass acceleration vector, $v^{\bar{\bar{\alpha ,\kappa }}}$ is the velocity of flow in an entity averaged over the boundary of the entity, $u_{i}^{\bar{\bar{\alpha ,\kappa }}}$ is the deviation velocity in an entity averaged over the boundary of the entity, $t^{\bar{\bar{\alpha }}}$ is the stress tensor, $\overset{\kappa \to \alpha }{T_{0}}$ represents the transfer of momentum from entity *κ* to entity *α*, and singular, or concentrated, forces of a common curve acting on a solid phase are included [19]. Conservation of energy equations is written for an overall entity as

(3) $$E_{**}^{\bar{\bar{\alpha }}}=\frac{{\text{D}}^{\bar{\alpha }}E^{\bar{\bar{\alpha }}}}{\text{D}t}+v^{\bar{\alpha }}\cdot \frac{{\text{D}}^{\bar{\alpha }}\left(\epsilon^{\bar{\bar{\alpha }}}\rho^{\alpha }v^{\bar{\alpha }}\right)}{\text{D}t}+\sum_{i\in J_{s}}\left(K_{E}^{\bar{\bar{i\alpha }}}+\frac{u^{\bar{\bar{i\alpha }}}\cdot u^{\bar{\bar{i\alpha }}}}{2}\right)\frac{{\text{D}}^{\bar{\alpha }}\left(\epsilon^{\bar{\bar{\alpha }}}\rho^{\alpha }\omega^{i\;\bar{\alpha }}\right)}{\text{D}t}\;-\frac{v^{\bar{\alpha }}\cdot v^{\bar{\alpha }}}{2}\sum_{i\in J_{s}}\frac{{\text{D}}^{\bar{\alpha }}\left(\epsilon^{\bar{\bar{\alpha }}}\rho^{\alpha }\omega^{i\;\bar{\alpha }}\right)}{\text{D}t}+\sum_{i\in J_{s}}\epsilon^{\bar{\bar{\alpha }}}\rho^{\alpha }\omega^{i\;\bar{\alpha }}\frac{{\text{D}}^{\bar{\alpha }}}{\text{D}t}\left(K_{E}^{\bar{\bar{i\alpha }}}+\frac{u^{\bar{\bar{i\alpha }}}\cdot u^{\bar{\bar{i\alpha }}}}{2}\right)\;+\left[E^{\bar{\bar{\alpha }}}+\epsilon^{\bar{\bar{\alpha }}}\rho^{\alpha }\frac{v^{\bar{\alpha }}\cdot v^{\bar{\alpha }}}{2}+\sum_{i\in J_{s}}\epsilon^{\bar{\bar{\alpha }}}\rho^{\alpha }\omega^{i\;\bar{\alpha }}\left(K_{E}^{\bar{\bar{i\alpha }}}+\frac{u^{\bar{\bar{i\alpha }}}\cdot u^{\bar{\bar{i\alpha }}}}{2}\right)\right]I:d^{\bar{\bar{\alpha }}}\;-\sum_{i\in J_{s}}\epsilon^{\bar{\bar{\alpha }}}\rho^{\alpha }\omega^{i\;\bar{\alpha }}g^{\bar{i\alpha }}\cdot \left(v^{\bar{\alpha }}+u^{\bar{\bar{i\alpha }}}\right)-\epsilon^{\bar{\bar{\alpha }}}h_{0}^{\bar{\bar{\alpha }}}-\epsilon^{\bar{\bar{\alpha }}}h^{\alpha }\;-\sum_{\kappa \in J_{c\alpha }}\sum_{i\in J_{s}}\overset{i\kappa \to i\alpha }{M}\left[{\bar{E}}_{i}^{\bar{\bar{\alpha ,\kappa }}}+\frac{\left(v^{\bar{\bar{\alpha ,\kappa }}}+u_{i}^{\bar{\bar{\alpha ,\kappa }}}\right)\cdot \left(v^{\bar{\bar{\alpha ,\kappa }}}+u_{i}^{\bar{\bar{\alpha ,\kappa }}}\right)}{2}+K_{Ei}^{\bar{\bar{\alpha ,\kappa }}}\right]\;-\sum_{\kappa \in J_{\text{c}\alpha }}\overset{\kappa \to \alpha }{T_{0}}\cdot v^{\bar{\bar{\alpha ,\kappa }}}-\sum_{\kappa \in J_{\text{c}\alpha }}\overset{\kappa \to \alpha }{Q_{1}}\;-\nabla \cdot \left(\epsilon^{\bar{\bar{\alpha }}}t^{\bar{\bar{\alpha }}}\cdot v^{\bar{\alpha }}+\epsilon^{\bar{\bar{\alpha }}}q^{\bar{\bar{\alpha }}}\right)=0\text{ for }\alpha \in J,$$

where $E^{\bar{\bar{\alpha }}}$ is the internal energy density, $K_{E}^{\bar{\bar{i\alpha }}}$ is the kinetic energy per unit mass resulting from velocity fluctuations, ${\bar{E}}_{i}^{\bar{\bar{\alpha ,\kappa }}}$ is the partial mass energy averaged over the boundary of the entity, $K_{Ei}^{\bar{\bar{\alpha ,\kappa }}}$ is the average deviation kinetic energy averaged over the boundary of the entity, $q^{\bar{\bar{\alpha }}}$ is the non-advective energy flux, *h*^*α*^ is the energy source density, $h_{0}^{\bar{\bar{\alpha }}}$ is the energy source density due to body force and velocity fluctuations, and $\overset{\kappa \to \alpha }{Q_{1}}$ is the transfer of internal energy from entity *κ* to entity *α* other than by phase change.

Equations (1)–(3) are the basic conservation equations needed to formulate the models of interest in this work. Additional, and available, equations needed for model simplification, closure, and completion will be introduced as needed in the formulation process.

## 5 Simplified entropy inequality

A key concept from the TCAT approach is the use of an entropy inequality to formulate closure relations that are consistent with the second law of thermodynamics. A strict flux–force form of the entropy inequality is needed to satisfy this purpose; this form is referred to as the SEI. The formulation of an SEI requires skill and substantial mathematical manipulations. However, once derived the SEI can be used without understanding all of the details needed to arrive at the final form. A general SEI is available for the class of model considered in this work [52] and can be written as

(4) $$\sum_{\alpha \in J_{\text{f}}}\frac{1}{\theta^{\bar{\bar{\alpha }}}}\left(\epsilon^{\bar{\bar{\alpha }}}t^{\bar{\bar{\alpha }}}+\epsilon^{\bar{\bar{\alpha }}}p^{\alpha }I\right):d^{\bar{\bar{\alpha }}}+\frac{1}{\theta^{\bar{\bar{s}}}}\left(\epsilon^{\bar{\bar{s}}}t^{\bar{\bar{s}}}-\epsilon^{\bar{\bar{s}}}t^{s}\right):d^{\bar{\bar{s}}}\;+\sum_{\alpha \in J_{I}}\frac{1}{\theta^{\bar{\bar{\alpha }}}}\left[\epsilon^{\bar{\bar{\alpha }}}t^{\bar{\bar{\alpha }}}-\epsilon^{\bar{\bar{\alpha }}}\gamma^{\alpha }\left(I-G^{\alpha }\right)\right]:d^{\bar{\bar{\alpha }}}\;+\frac{1}{\theta^{\bar{\bar{wns}}}}\left[\epsilon^{\bar{\bar{wns}}}t^{\bar{\bar{wns}}}+\epsilon^{\bar{\bar{wns}}}\gamma^{wns}\left(I-G^{wns}\right)\right]:d^{\bar{\bar{wns}}}\;-\sum_{\alpha \in J}\left[\epsilon^{\bar{\bar{\alpha }}}q^{\bar{\bar{\alpha }}}+\epsilon^{\bar{\bar{\alpha }}}q_{g0}^{\bar{\bar{\alpha }}}+\sum_{i\in J_{s}}\epsilon^{\bar{\bar{\alpha }}}\rho^{\alpha }\omega^{i\;\bar{\alpha }}\left(\mu^{\bar{i\alpha }}+K_{E}^{\bar{\bar{i\alpha }}}+\frac{u^{\bar{\bar{i\alpha }}}\cdot u^{\bar{\bar{i\alpha }}}}{2}\right)u^{\bar{\bar{i\alpha }}}\right]\cdot \nabla \left(\frac{1}{\theta^{\bar{\bar{\alpha }}}}\right)\;-\sum_{\alpha \in J}\sum_{i\in J_{s}\backslash N}\frac{1}{\theta^{\bar{\bar{\alpha }}}}\epsilon^{\bar{\bar{\alpha }}}\rho^{\alpha }\omega^{i\;\bar{\alpha }}u^{\bar{i\alpha }}\cdot \nabla [\mu^{\bar{i\alpha }}+K_{E}^{\bar{\bar{i\alpha }}}+\frac{u^{\bar{\bar{i\alpha }}}\cdot u^{\bar{\bar{i\alpha }}}}{2}+\psi^{\bar{i\alpha }}\;-\left(\mu^{\bar{N\alpha }}+K_{E}^{\bar{\bar{N\alpha }}}+\frac{u^{\bar{\bar{N\alpha }}}\cdot u^{\bar{N\alpha }}}{2}+\psi^{\bar{N\alpha }}\right)]\;-\sum_{\alpha \in J}\sum_{i\in J_{s}}\frac{1}{\theta^{\bar{\bar{\alpha }}}}\left(\mu^{\bar{i\alpha }}+K_{E}^{\bar{\bar{i\alpha }}}+\frac{u^{\bar{\bar{i\alpha }}}\cdot u^{\bar{\bar{i\alpha }}}}{2}+\psi^{\bar{i\alpha }}\right)\epsilon^{\bar{\bar{\alpha }}}r^{i\alpha }\;+\sum_{\alpha \in J}\sum_{i\in J_{s}}\frac{1}{\theta^{\bar{\bar{\alpha }}}}{\langle r_{i\alpha }\psi_{i\alpha }\rangle }_{\Omega_{\alpha },\Omega }\;+\sum_{\alpha \in J_{\text{f}}}\sum_{\kappa \in J_{\text{c}\alpha }}\sum_{i\in J_{s}}\overset{i\alpha \to i\kappa }{M}[\frac{1}{\theta^{\bar{\bar{\alpha }}}}\left(\mu^{\bar{i\alpha }}+K_{E}^{\bar{\bar{i\alpha }}}+\frac{u^{\bar{\bar{i\alpha }}}\cdot u^{\bar{\bar{i\alpha }}}}{2}+\psi^{\bar{i\alpha }}\right)\;-\frac{1}{\theta^{\bar{\bar{\kappa }}}}\left(\mu^{\bar{i\kappa }}+K_{E}^{\bar{\bar{i\kappa }}}+\frac{u^{\bar{\bar{i\kappa }}}\cdot u^{\bar{\bar{i\kappa }}}}{2}+\psi^{\bar{i\kappa }}\right)]\;+\sum_{\kappa \in J_{cs}}\sum_{i\in J_{s}}\overset{is\to i\kappa }{M}\left[\frac{1}{\theta^{\bar{\bar{s}}}}\left(\mu^{\bar{is}}+K_{E}^{\bar{\bar{is}}}+\frac{u^{\bar{\bar{is}}}\cdot u^{\bar{\bar{is}}}}{2}+\psi^{\bar{is}}+\frac{\sigma^{\bar{\bar{s}}}:C^{s}}{3\rho^{s}j^{s}}\right)\right.\;\left.-\frac{1}{\theta^{\bar{\bar{\kappa }}}}\left(\mu^{\bar{i\kappa }}+K_{E}^{\bar{\bar{i\kappa }}}+\frac{u^{\bar{\bar{i\kappa }}}\cdot u^{\bar{\bar{i\kappa }}}}{2}+\psi^{\bar{i\kappa }}\right)\right]\;+\sum_{\alpha \in J_{\text{I}}}\sum_{i\in J_{s}}\overset{i\alpha \to iwns}{M}[\frac{1}{\theta^{\bar{\bar{\alpha }}}}\left(\mu^{\bar{i\alpha }}+K_{E}^{\bar{\bar{i\alpha }}}+\frac{u^{\bar{\bar{i\alpha }}}\cdot u^{\bar{\bar{i\alpha }}}}{2}+\psi^{\bar{i\alpha }}\right)\;-\frac{1}{\theta^{\bar{\bar{wns}}}}\left(\mu^{\bar{iwns}}+K_{E}^{\bar{\bar{iwns}}}+\frac{u^{\bar{\bar{iwns}}}\cdot u^{\bar{\bar{iwns}}}}{2}+\psi^{\bar{iwns}}\right)]\;-\sum_{\alpha \in J_{\text{f}}}\{\overset{\alpha \to wn}{Q_{1}}+\overset{\alpha \to wn}{G_{0}}+\sum_{i\in J_{s}}\left({\bar{E}}_{i\alpha }^{\bar{wn}}+K_{Ei\alpha }^{\bar{\bar{wn}}}+\frac{u_{i\alpha }^{\bar{\bar{wn}}}\cdot u_{i\alpha }^{\bar{\bar{un}}}}{2}+\psi_{i\alpha }^{\bar{wn}}\right)\overset{i\alpha \to iwn}{M}\;+\left[\overset{\alpha \to wn}{T_{0}}+\sum_{i\in J_{s}}\left(\frac{v_{\alpha }\bar{\bar{\omega n}}-v^{\bar{s}}}{2}+u_{i\alpha }^{\bar{\bar{wn}}}\right)\overset{i\alpha \to iwn}{M}\right]\cdot \left(v_{\alpha }^{\bar{\bar{wn}}}-v^{\bar{s}}\right)\;-\left(\frac{{\text{D}}^{\bar{s}}\epsilon^{\bar{\bar{\alpha }}}}{\text{D}t}-\chi_{s}^{\bar{\bar{\alpha s}}}\frac{{\text{D}}^{\bar{s}}\epsilon }{\text{D}t}\right)p_{\alpha }^{wn}\}\left(\frac{1}{\theta^{\bar{\bar{\alpha }}}}-\frac{1}{\theta^{\bar{\bar{wn}}}}\right)\;-\sum_{\alpha \in J_{\text{f}}}\{\overset{\alpha \to \alpha s}{Q_{1}}+\overset{\alpha \to \alpha s}{G_{0}}+\sum_{i\in J_{s}}\left({\bar{E}}_{i\alpha }^{\bar{\alpha s}}+K_{Ei\alpha }^{\bar{\bar{\alpha s}}}+\frac{u_{i\alpha }^{\bar{\bar{\alpha s}}}\cdot u_{i\alpha }^{\bar{\bar{\alpha s}}}}{2}+\psi_{i\alpha }^{\bar{\alpha s}}\right)\overset{i\alpha \to i\alpha s}{M}\;+\left[\overset{\alpha \to \alpha s}{T_{0}}+\sum_{i\in J_{s}}\left(\frac{v_{\alpha }^{\bar{\bar{\alpha s}}}-v^{\bar{s}}}{2}+u_{i\alpha }^{\bar{\bar{\alpha s}}}\right)\overset{i\alpha \to i\alpha s}{M}\right]\cdot \left(v_{\alpha }^{\bar{\alpha s}}-v^{\bar{s}}\right)\;-\chi_{s}^{\bar{\bar{\alpha s}}}\frac{{\text{D}}^{\bar{s}}\epsilon }{\text{D}t}p_{\alpha }^{\alpha s}\}\left(\frac{1}{\theta^{\bar{\bar{\alpha }}}}-\frac{1}{\theta^{\bar{\bar{\alpha s}}}}\right)\;-\sum_{\kappa \in J_{cs}^{-}}\{\overset{s\to \kappa }{Q_{1}}+\overset{s\to \kappa }{G_{0}}+\sum_{i\in J_{s}}\left({\bar{E}}_{is}^{\bar{\kappa }}+K_{Eis}^{\bar{\bar{\kappa }}}+\frac{u_{is}^{\bar{\bar{\kappa }}}\cdot u_{is}^{\bar{\bar{\kappa }}}}{2}+\psi_{is}^{\bar{\kappa }}\right)\overset{is\to i\kappa }{M}\;+\left[\overset{s\to \kappa }{T_{0}}+\sum_{i\in J_{s}}\left(\frac{v_{s}^{\bar{\bar{\kappa }}}-v^{\bar{s}}}{2}+u_{is}^{\bar{\bar{\kappa }}}\right)\overset{is\to i\kappa }{M}\right]\cdot \left(v_{s}^{\bar{\bar{\kappa }}}-v^{\bar{s}}\right)\;-\chi_{s}^{\bar{\bar{\kappa }}}\frac{{\text{D}}^{\bar{s}}\epsilon }{\text{D}t}{\left(n_{s}\cdot t_{s}\cdot n_{s}\right)}_{s}^{\kappa }\}\left(\frac{1}{\theta^{\bar{\bar{s}}}}-\frac{1}{\theta^{\bar{\bar{\kappa }}}}\right)\;-\{\overset{wn\to wns}{Q_{1}}+\overset{wn\to wns}{G_{0}}+\sum_{i\in J_{s}}\left({\bar{E}}_{iwn}^{\bar{wns}}+K_{Eiwn}^{\bar{\bar{wns}}}+\frac{u_{iwn}^{\bar{\bar{wns}}}\cdot u_{iwn}^{\bar{\bar{\bar{wns}}}}}{2}+\psi_{iwn}^{\bar{wns}}\right)\overset{iwn\to iwns}{M}\;+\left[\overset{wn\to wns}{T_{0}}+\sum_{i\in J_{s}}\left(\frac{v_{wn}^{\bar{\bar{wns}}}-v^{\bar{s}}}{2}+u_{iwn}^{\bar{\bar{wns}}}\right)\overset{iwn\to iwns}{M}\right]\cdot \left(v_{wn}^{\bar{\bar{wns}}}-v^{\bar{s}}\right)\;+[\left(\epsilon^{\bar{\bar{ws}}}+\epsilon^{\bar{\bar{ns}}}\right)\frac{{\text{D}}^{\bar{s}}\chi_{s}^{\bar{\bar{ws}}}}{\text{D}t}\text{cos }\varphi^{\bar{\bar{ws,wn}}}\;+\frac{\epsilon^{\bar{\bar{wns}}}}{\epsilon^{\bar{\bar{ws}}}+\epsilon^{\bar{\bar{ns}}}}\frac{{\text{D}}^{\bar{s}}\epsilon }{\text{D}t}\text{sin }\varphi^{\bar{\bar{ws,wn}}}]\gamma_{wn}^{wns}\}\left(\frac{1}{\theta^{\bar{\bar{wn}}}}-\frac{1}{\theta^{\bar{\bar{wns}}}}\right)\;-\sum_{\alpha \in J_{\text{f}}}\{\overset{\alpha s\to wns}{Q_{1}}+\overset{\alpha s\to wns}{G_{0}}+\sum_{i\in J_{s}}\left({\bar{E}}_{i\alpha s}^{\bar{wns}}+K_{Ei\alpha s}^{\bar{\bar{wns}}}+\frac{u_{i\alpha s}^{\bar{\bar{wns}}}\cdot u_{i\alpha s}^{\bar{\bar{wns}}}}{2}+\psi_{i\alpha s}^{\bar{wns}}\right)\overset{i\alpha s\to iwns}{M}\;+\left[\overset{\alpha s\to wns}{T_{0}}+\sum_{i\in J_{s}}\left(\frac{v_{\alpha s}^{\bar{\bar{wns}}}-v^{\bar{s}}}{2}+u_{i\alpha s}^{\bar{\bar{wns}}}\right)\overset{i\alpha s\to iwns}{M}\right]\cdot \left(v_{\alpha s}^{\bar{\bar{wns}}}-v^{\bar{s}}\right)\;+\left(\epsilon^{\bar{\bar{ws}}}+\epsilon^{\bar{\bar{ns}}}\right)\frac{{\text{D}}^{\bar{s}}\chi_{s}^{\bar{\bar{\alpha s}}}}{\text{D}t}\gamma_{\alpha s}^{wns}\}\left(\frac{1}{\theta^{\bar{\bar{\alpha s}}}}-\frac{1}{\theta^{\bar{\bar{wns}}}}\right)\;-\overset{s\to wns}{Q_{1}^{*}}\left(\frac{1}{\theta^{\bar{\bar{s}}}}-\frac{1}{\theta^{\bar{\bar{wns}}}}\right)\;-\sum_{\alpha \in J_{\text{f}}}\frac{1}{\theta^{\bar{\bar{\alpha }}}}\{\eta^{\bar{\bar{\alpha }}}\nabla \theta^{\bar{\bar{\alpha }}}-\nabla \left(\epsilon^{\bar{\bar{\alpha }}}p^{\alpha }\right)\;+\sum_{i\in J_{s}}\epsilon^{\bar{\bar{\alpha }}}\rho^{\alpha }\omega^{i\;\bar{\alpha }}\left[\nabla \left(\mu^{\bar{i\alpha }}+K_{E}^{\bar{\bar{i\alpha }}}+\frac{u^{\bar{\bar{i\alpha }}}\cdot u^{\bar{\bar{i\alpha }}}}{2}+\psi^{\bar{i\alpha }}\right)+g^{\bar{i\alpha }}\right]\;-\sum_{\kappa \in J_{c\alpha }}[\overset{\alpha \to \kappa }{T_{0}}-\sum_{i\in J_{s}}\frac{\left(v^{\bar{\alpha }}-v^{\bar{s}}\right)}{2}\overset{i\alpha \to i\kappa }{M}\;+\sum_{i\in J_{s}}\left(v_{\alpha }^{\bar{\bar{\kappa }}}+u_{i\alpha }^{\bar{\bar{\kappa }}}-v^{\bar{s}}\right)\overset{i\alpha \to i\kappa }{M}]\}\cdot \left(v^{\bar{\alpha }}-v^{\bar{s}}\right)\;-\sum_{\alpha \in J_{I}}\frac{1}{\theta^{\bar{\bar{\alpha }}}}\{\eta^{\bar{\bar{\alpha }}}\left(I-G^{\alpha }\right)\cdot \nabla \theta^{\bar{\bar{\alpha }}}+\nabla \cdot \left[\left(I-G^{\alpha }\right)\epsilon^{\bar{\bar{\alpha }}}\gamma^{\alpha }\right]\;+\sum_{i\in J_{s}}\epsilon^{\bar{\bar{\alpha }}}\rho^{\alpha }\omega^{i\;\bar{\alpha }}\left(I-G^{\alpha }\right)\cdot \nabla \left(\mu^{\bar{i\alpha }}+K_{E}^{\bar{\bar{i\alpha }}}+\frac{u^{\bar{\bar{i\alpha }}}\cdot u^{\bar{\bar{i\alpha }}}}{2}+\psi^{\bar{i\alpha }}\right)+\sum_{i\in J_{s}}\epsilon^{\bar{\bar{\alpha }}}\rho^{\alpha }\omega^{i\;\bar{\alpha }}g^{\bar{i\alpha }}\;+\sum_{\kappa \in J_{c\alpha }^{+}}\left[\overset{\kappa \to \alpha }{T_{0}}-\sum_{i\in J_{s}}\frac{\left(v^{\bar{\alpha }}-v^{\bar{s}}\right)}{2}\overset{i\kappa \to i\alpha }{M}+\sum_{i\in J_{s}}\left(v_{\kappa }^{\bar{\bar{\alpha }}}+u_{i\kappa }^{\bar{\bar{\alpha }}}-v^{\bar{s}}\right)\overset{i\kappa \to i\alpha }{M}\right]\;-[\overset{\alpha \to wns}{T_{0}}-\sum_{i\in J_{s}}\frac{\left(v^{\bar{\alpha }}-v^{\bar{s}}\right)}{2}\overset{i\alpha \to iwns}{M}\;+\sum_{i\in J_{s}}\left(v_{\alpha }^{\bar{\bar{wns}}}+u_{i\alpha }^{\bar{\bar{wns}}}-v^{\bar{s}}\right)\overset{i\alpha \to iwns}{M}]\}\cdot \left(v^{\bar{\alpha }}-v^{\bar{s}}\right)\;-\frac{1}{\theta^{\bar{\bar{wns}}}}\{\eta^{\bar{\bar{wns}}}\left(I-G^{wns}\right)\cdot \nabla \theta^{\bar{\bar{wns}}}-\nabla \cdot \left[\left(I-G^{wns}\right)\epsilon^{\bar{\bar{wns}}}\gamma^{wns}\right]\;+\sum_{i\in J_{s}}\epsilon^{\bar{\bar{wns}}}\rho^{wns}\omega^{i\;\bar{wns}}\left(I-G^{wns}\right)\cdot \nabla \left(\mu^{\bar{iwns}}+K_{E}^{\bar{\bar{iwns}}}+\frac{u^{\bar{\bar{iwns}}}\cdot u^{\bar{\bar{iwns}}}}{2}+\psi^{\bar{iwns}}\right)\;+\sum_{i\in J_{s}}\epsilon^{\bar{\bar{wns}}}\rho^{wns}\omega^{i\;\bar{wns}}g^{\bar{iwns}}\;+\sum_{\kappa \in J_{\text{c}wns}^{+}}[\overset{\kappa \to wns}{T_{0}}-\sum_{i\in J_{s}}\frac{\left(v^{\bar{wns}}-v^{\bar{s}}\right)}{2}\overset{i\kappa \to iwns}{M}\;+\sum_{i\in J_{s}}\left(v_{\kappa }^{\bar{\bar{wns}}}+u_{i\kappa }^{\bar{\bar{wns}}}-v^{\bar{s}}\right)\overset{i\kappa \to iwns}{M}]\;-{\langle n_{s}\cdot t_{s}^{*}\cdot n_{s}n_{s}\rangle }_{\Omega_{wns},\Omega }\}\cdot \left(v^{\bar{wns}}-v^{\bar{s}}\right)\;+\frac{1}{\theta^{\bar{\bar{wn}}}}\left[\frac{{\text{D}}^{\bar{s}}\epsilon^{\bar{\bar{w}}}}{\text{D}t}-\chi_{s}^{\bar{\bar{ws}}}\frac{{\text{D}}^{\bar{s}}\epsilon }{\text{D}t}-\frac{\gamma^{wn}{\hat{k}}_{1}^{wn}\left(\epsilon^{\bar{\bar{wn}}}-\epsilon_{\text{eq}}^{\bar{\bar{wn}}}\right)}{p_{w}^{wn}-p_{n}^{wn}+\sum_{i\in J_{s}}\rho^{wn}\omega^{i\;\bar{wn}}{\left(g_{iwn}\cdot n_{w}\right)}^{\bar{wn}}}\right]\;\times \left(p_{w}^{wn}-p_{n}^{wn}+\sum_{i\in J_{s}}\rho^{wn}\omega^{i\;\bar{wn}}{\left({\text{g}}_{iwn}\cdot n_{w}\right)}^{\bar{wn}}-\gamma^{wn}J_{w}^{wn}\right)\;+\frac{1}{\theta^{\bar{\bar{ws}}}}\chi_{s}^{\bar{\bar{ws}}}\frac{{\text{D}}^{\bar{s}}\epsilon }{\text{D}t}\left[p_{w}^{ws}+{\left(n_{s}\cdot t_{s}\cdot n_{s}\right)}_{s}^{ws}-\sum_{i\in J_{s}}\rho^{ws}\omega^{i\;\bar{ws}}{\left(g_{iws}\cdot n_{s}\right)}^{\bar{ws}}+\gamma^{ws}J_{s}^{ws}\right]\;+\frac{1}{\theta^{\bar{\bar{ns}}}}\chi_{s}^{\bar{\bar{ns}}}\frac{{\text{D}}^{\bar{s}}\epsilon }{\text{D}t}\left[p_{n}^{ns}+{\left(n_{s}\cdot t_{s}\cdot n_{s}\right)}_{s}^{ns}-\sum_{i\in J_{s}}\rho^{ns}\omega^{i\;\bar{ns}}{\left(g_{ins}\cdot n_{s}\right)}^{\bar{ns}}+\gamma^{ns}J_{s}^{ns}\right]\;-\frac{1}{\theta^{\bar{\bar{wns}}}}\left(\epsilon^{\bar{\bar{ws}}}+\epsilon^{\bar{\bar{ns}}}\right)\frac{{\text{D}}^{\bar{s}}\chi_{s}^{\bar{\bar{ws}}}}{\text{D}t}[\gamma_{wn}^{wns}\text{cos }\varphi^{\bar{\bar{ws,wn}}}+\gamma_{ws}^{wns}-\gamma_{ns}^{wns}\;+\gamma^{wns}\kappa_{G}^{\bar{\bar{wns}}}-\sum_{i\in J_{s}}\rho^{wns}\omega^{i\;\bar{wns}}{\left(g_{iwns}\cdot n_{ws}\right)}^{\bar{wns}}]\;-\frac{1}{\theta^{\bar{\bar{wns}}}}\left(\frac{\epsilon^{\bar{\bar{wns}}}}{\epsilon^{\bar{\bar{ws}}}+\epsilon^{\bar{\bar{ns}}}}\right)\frac{D^{\bar{s}}\epsilon }{Dt}[\gamma_{wn}^{wns}\text{sin }\varphi^{\bar{\bar{ws,wn}}}-\gamma^{wns}\kappa_{N}^{\bar{\bar{wns}}}\;-{\langle n_{s}\cdot t_{s}^{*}\cdot n_{s}\rangle }_{\Omega_{wns},\Omega_{wns}}+\sum_{i\in J_{s}}\rho^{wns}\omega^{i\;\bar{wns}}{\left(g_{iwns}\cdot n_{s}\right)}^{\bar{wns}}]\;=\sum_{\alpha \in J}\Lambda^{\bar{\bar{\alpha }}}\geq 0,$$

where all symbols are defined in the notation section and the interactions between the solid phase and the common curve have been explicitly noted.

The general SEI given by Eq. (4) contains the flux–force pairs that can produce entropy in a system. While they may appear overwhelming to the non-specialist, a brief review of the terms by line number can aid understanding of the fluxes considered for which closure relations will be developed. Lines 1–3 are fluxes involving stress tensors and their products with deformation rate tensors. Line 4 is a conductive heat transfer and deviation term flux and their product with a temperature gradient force. Lines 5 and 6 consist of a deviation velocity flux and a force that is a gradient in potential terms, and higher order terms in deviation velocities. Lines 7 and 8 are reaction fluxes resulting from potentials and deviation quantities grouped as forces. Lines 9–14 represent inter-entity mass transfer resulting from differences in potentials and deviation quantities. Lines 18–31 express the inter-entity transfer of energy resulting from forces that are a difference in temperatures. Lines 32–46 are momentum fluxes resulting from differences in entity velocities. Lines 47 and 48 are fluxes in extent measures resulting from a deviation in capillary pressure between the fluid phases from equilibrium. Lines 49 and 50 are changes in porosity resulting from a balance of forces in the direction normal to the solid phase. Lines 51 and 52 represent a change in the wetted fraction of the solid phase resulting from motion of the common curve due to a balance of forces acting tangent to the surface of the solid phase, Lines 53 and 54 express the changes in the porosity resulting from common curve forces acting normal to the solid surface, and Line 55 is the total entropy density production rate of the system. Because each member of the set of fluxes is independent of all other members of the set of fluxes, the fluxes can be considered in turn to derive permissible forms of the closure relations. Examples of how to do so are available in the literature [19,28,52]; examples for modeling tumors are detailed in the following sections.

## 6 Two-phase system

### 6.1 Description

The purpose of this section is to consider a relatively simple macroscale model to describe tumor growth. This example will enable a straightforward exposition demonstrating the TCAT model building and closure process. Since the focus is on the model formulation process, neither the model solution nor evaluation will be considered herein.

We wish to use the TCAT model components summarized above to formulate a macroscale model consisting of two phases: a solid phase denoted with the index *s*, and a single fluid phase denoted with the index *f*, which is a subset of the more general two-fluid phase TCAT model hierarchy that is summarized above. The solid phase consists of the extra-cellular matrix (ECM), live and necrotic tumor cells, glucose, oxygen, water, and a chemotherapeutic drug. These species are important for the solid phase because of the separation of mass transfer and homogeneous phase reactions, and the conceptual representation of the operative processes affecting tumor growth. The fluid phase represents the interstitial fluid, which consists of a dominant water species, glucose, oxygen, and a chemotherapeutic drug. Both phases contain a background species that comprises all other species that are not explicitly considered. Lysis of necrotic cells is also considered.

Based on the above description, the index set of entities is

(5) J={f,s,fs},

where *fs* denotes the fluid–solid interface.

The index set of the nine species considered is denoted

(6) $$J_{s}=\left\{l,n,e,g,o,c,w,x,y\right\},$$

where *l* denotes a living tumor species, *n* a necrotic tumor species, *e* the extra-cellular matrix species, *g* glucose, *o* oxygen, *c* a chemotherapeutic drug, *w* the water, *x* a collective background species for the fluid phase, and *y* a collective background species for the solid phase. The background species represent a set of non-reactive background species that do not undergo mass transfer. The solid phase may contain all species but *x*, and the fluid phase may contain the *g, o, c, w* and *x* species.

Primary restrictions specify the general basis for the TCAT model framework to be used. The primary restrictions for the model are: continuum mechanics represents the system of concern with sufficient fidelity; a clear separation of length scales exists between the microscale and the macroscale; and classical irreversible thermodynamics can be used to describe the system of concern. Secondary restrictions are specified to simplify the general TCAT model hierarchy to the simplest possible form that represents the system of concern. These restrictions have implications for model formulation and closure, including simplification of the general SEI. The secondary restrictions for this application are: (SR1-2P) the system consists of one fluid phase, one solid phase, and an interface; (SR2-2P) the system is isothermal; (SR3-2P) the interface is massless, (SR4-2P) kinetic energy terms are higher order and of negligible importance; (SR5-2P) body force vectors and potentials are identical for all species; (SR6-2P) chemical reactions can be formulated in terms of chemical affinities; (SR7-2P) inertial terms in the momentum equations are insignificant due to the slow dynamics of the systems considered; (SR8-2P) density-weighted, area-averaged velocities and deviation velocities are equal to their volume-averaged counterparts; (SR9-2P) the Lagrangian stress tensor product with the Green’s deformation tensor can be neglected from the driving force difference for mass transfer to the solid phase; and (SR10-2P) the activity coefficient of all species is unity.

### 6.2 Formulation

The two-phase model described above will be formulated into a closed mathematical model. The steps involved in doing so are detailed in the subsections that follow and include the formulation of a restricted SEI, use of the restricted SEI to formulate a permissible set of closure relations, and a closed model formulation based upon conservation equations and closure approximations.

#### 6.2.1 Restricted SEI

Equation (4) is a general expression that applies to a wide range of systems involving two fluid phases, a solid phase, three interfaces, and a common curve; and a set of flux–force pairs involving dissipative processes involving mass, momentum, and energy. This general expression can be simplified for the example two-phase system described above, and many other applications of concern as well. While several general SEIs have been developed for various systems [16–18,28,29,52], the ability to simplify a general SEI for a complex system to describe simpler systems involving fewer entities than the general expression is a hallmark of the TCAT approach. For the application at hand, the original seven entity SEI can be reduced to a three entity SEI, where J={f,s,fs}. Furthermore, the other secondary restrictions noted above result in substantial simplifications of Eq. (4), while preserving the necessary terms needed to formulate a closed, well-posed model. Applying all secondary restrictions to Eq. (4) yields the restricted SEI given by

(7) $$\frac{1}{\theta }\left(\epsilon^{\bar{\bar{f}}}t^{\bar{\bar{f}}}+\epsilon^{\bar{\bar{f}}}p^{f}I\right):d^{\bar{\bar{f}}}+\frac{1}{\theta }\left(\epsilon^{\bar{\bar{s}}}t^{\bar{\bar{s}}}-\epsilon^{\bar{\bar{s}}}t^{s}\right):d^{\bar{\bar{s}}}\;+\frac{1}{\theta }\left[\epsilon^{\bar{\bar{fs}}}t^{\bar{\bar{fs}}}-\epsilon^{\bar{\bar{fs}}}\gamma^{fs}\left(I-G^{fs}\right)\right]:d\bar{\bar{fs}}\;-\sum_{\alpha \in J_{\text{P}}}\sum_{i\in J_{s}\backslash N}\frac{1}{\theta }\epsilon^{\bar{\bar{\alpha }}}\rho^{\alpha }\omega^{i\;\bar{\alpha }}u^{\bar{\bar{i\alpha }}}\cdot \nabla \left(\mu^{\bar{i\alpha }}-\mu^{\bar{N\alpha }}\right)\;-\sum_{\alpha \in J_{\text{P}}}\sum_{k\in J_{\text{rxn}\alpha }}\frac{1}{\theta }\epsilon^{\bar{\bar{\alpha }}}R^{k\alpha }A^{k\alpha }\;+\sum_{i\in J_{s}}\overset{if\to is}{M}\frac{1}{\theta }\left(\mu^{\bar{if}}+\psi^{\bar{f}}-\mu^{\bar{is}}-\psi^{\bar{s}}\right)\;-\frac{1}{\theta }\{-\nabla \left(\epsilon^{\bar{\bar{f}}}p^{f}\right)+\epsilon^{\bar{\bar{f}}}\rho^{f}\left(\sum_{i\in J_{s}}\omega^{i\;\bar{f}}\nabla \mu^{\bar{if}}+\nabla \psi^{\bar{f}}+g^{\bar{f}}\right)\;-\overset{f\to fs}{T_{0}}\}\cdot \left(v\bar{f}-v^{\bar{s}}\right)\;-\frac{1}{\theta }\left\{\nabla \cdot \left[\left(I-G^{fs}\right)\epsilon^{\bar{\bar{fs}}}\gamma^{fs}\right]+\overset{f\to fs}{T_{0}}+\overset{s\to fs}{T_{0}}\right\}\cdot \left(v^{\bar{fs}}-v^{\bar{s}}\right)\;+\frac{1}{\theta }\frac{{\text{D}}^{\bar{s}}\epsilon }{\text{D}t}\left[p_{f}^{fs}+{\left(n_{s}\cdot t_{s}\cdot n_{s}\right)}_{s}^{fs}+\gamma^{fs}J_{s}^{fs}\right]\;=\sum_{\alpha \in J}\Lambda^{\bar{\bar{\alpha }}}\geq 0,$$

where *θ* is the isothermal temperature of the system, $J_{\text{rxn}\alpha }$ is the index set of all reaction equations in the *α* phase, *R*^*kα*^ is a molar rate of reaction, *A*^*kα*^ is a chemical affinity, and SR6-2P has been used to write the reactions in terms of chemical affinities [19]. Each of these flux–force pairs is explained physically and used to formulate a permissible set of closure relations in the section that follows.

#### 6.2.2 Closure relations

We will consider the flux–force pairs in Eq. (7) in order and use these pairs to formulate a permissible set of closure relations, which will be used to formulate a closed, solvable model. References will be made to line numbers in this equation as individual flux–force pairs are considered. Each member of the set of fluxes is independent of all other fluxes, and each member of the set of forces is independent of all other forces. However, a flux may depend upon not only the conjugate force that appears as product in the SEI but also on other members of the set of forces as well, which is referred to as cross-coupled closure. The SEI is an equation that specifies constraints on permissible forms of closure relations. For both of these reasons, closure relations are not unique, and the complexity of the approximate forms can be tailored to the applications. Approaches to derive approximations have been developed and found to have utility for describing a range of physical systems [19]. We will follow these traditional approaches to generate models that can be compared to data. If the models are not consistent with the observations, the closure relation approximations and the model restrictions may be reexamined and modified as needed. Thus, a clear path to modifying macroscale continuum models exists.

The first term in Line 1 of Eq. (7) represents a flux involving the stress tensor for the fluid and the fluid pressure, and the conjugate force is the deformation rate tensor for the fluid. The product of this flux and force must be nonnegative under all conditions and zero at equilibrium. A zero-order approximation is the simplest possible approximation, and it has been found to provide a good description of macroscale porous medium systems [19]. The zero-order approximation is that the flux term is zero under all conditions such that

(8) $$t^{\bar{\bar{f}}}+p^{f}I=0,$$

allowing the macroscale stress tensor to be approximated as

(9) $$t^{\bar{\bar{f}}}=-p^{f}I.$$

This is a reasonable approximation because at the macroscale fluid flow through a porous medium is essentially inviscid, with momentum transfer to the solid phase particles being a dominant process. This dominant exchange process is represented by $\overset{f\to fs}{T_{0}}$, which appears in Line 7 and is discussed below.

Following a similar line of reasoning, the stress tensor for the solid phase is

(10) $$t^{\bar{\bar{s}}}=t^{s},$$

and the stress tensor for the interface appearing in Line 2 of Eq. (7) is

(11) $$t^{\bar{\bar{fs}}}=\gamma^{fs}\left(I-G^{fs}\right).$$

Equation (10) suggests that the macroscale stress tensor for the solid phase can be derived based upon intrinsic averaging of the microscale stress tensor, which is determinate based upon the solid behavior at the microscale. Equation (11) relates the interfacial stress tensor to the product of the interfacial tension and the orientation of the interface.

Line 3 in Eq. (7) involves a flux represented by the deviation velocity $u^{\bar{\bar{i\alpha }}}$ and the conjugate force, which is the gradient of the chemical potential. The difference in chemical potential occurs because of the necessary constraint that

(12) $$\sum_{i\in J_{s}}\omega^{i\;\bar{\alpha }}u^{\bar{\bar{i\alpha }}}=0\text{ for }\alpha \in J,$$

which implies that *N −* 1 deviation velocities are independent. Choosing *w* as the dominant reference species in the *f* phase and *e* as the dominant reference species in the *s* phase yields the linear first-order closure relation for the fluid phase given by

(13) $$\omega^{i\;\bar{f}}u^{\bar{\bar{if}}}=-{\hat{D}}_{iw}^{f}\cdot \nabla \left(\mu^{\bar{if}}-\mu^{\bar{wf}}\right)\text{ for }i\in \left\{g,o,c,x\right\},$$

and the corresponding closure relation for the solid-phase deviation velocity given by

(14) $$\omega^{i\;\bar{s}}u^{\bar{\bar{is}}}=-{\hat{D}}_{ie}^{s}\cdot \nabla \left(\mu^{\bar{is}}-\mu^{\bar{es}}\right)\text{ for }i\in \left\{l,n,g,o,c,w,y\right\},$$

where ${\hat{D}}_{ij}^{\alpha }$ are second-rank symmetric tensors that parameterize the deviation velocities in terms of gradients in chemical potentials.

Line 4 of Eq. (7) expresses the flux–force pair of the rate of reaction and the chemical activity. A permissible closure relation must be generated from this pair and related to the reaction term appearing in Eq. (1), which is the term involving *r*^*iα*^ that is the rate of mass production of species *i* per time resulting from all reactions in phase *α*. A linear closure relation is

(15) $$R^{k\alpha }=-{\hat{K}}_{k\alpha }A^{k\alpha }\text{ for }\alpha \in J_{\text{P}},$$

where ${\hat{K}}_{k\alpha }$ is a nonnegative reaction rate coefficient for the *k*^*th*^ reaction in the *α* phase. Substitution of this approximation into the SEI yields a nonnegative production rate.

Two tasks remain to formulate closure approximations for *r*^*iα*^: the closure approximation must be related to the general reaction variable, and the set of chemical reactions occurring in each phase must be formulated. The overall mass production rate results from the set of reactions such that

(16) $$r^{i\alpha }=\underset{k\in J_{\text{rxn}\alpha }}{\sum }v_{ik\alpha }MW_{i}R^{k\alpha }\text{ for }\alpha \in J_{\text{P}},$$

and the affinity is defined as

(17) $$A^{k\alpha }=\underset{i\in J_{s}}{\sum }\mu^{\bar{i\alpha }}v_{ik\alpha }MW_{i}\text{ for }k\in J_{\text{rxn}\alpha },\alpha \in J_{\text{P}},$$

where *MW* is the molecular weight, and *ν* is a molar stoichiometric reaction coefficient. Equations (15)–(17) can be combined to yield the mass production rate resulting from reaction given by

(18) $$r^{i\alpha }=-\underset{k\in J_{\text{rxn}\alpha }}{\sum }{\hat{K}}_{k\alpha }\nu_{ik\alpha }MW_{i}\left(\underset{i\in J_{s}}{\sum }\mu^{\bar{i\alpha }}v_{ik\alpha }MW_{i}\right)\text{ for }\alpha \in J_{\text{p}}.$$

If the set of molar biochemical reactions and molecular weights are known for all reactions in each phase, then the species mass production rate is fully specified and closed. When the reactions are nonlinear, resolution issues related to averaging exist, which will not be considered herein [19].

It is assumed that no reactions occur in the fluid phase, which implies that the *g, c*, and *o* species are non-reactive in this phase, although all species may undergo mass transfer to and from the solid phase. Reactions occur in the solid phase that lead to tumor growth, the consumption of glucose, oxygen and the chemotherapeutic drug species, tumor species death due to the chemotherapy drug, conversion of a living tumor to a necrotic tumor resulting from a lack of oxygen, and necrotic tumor lysis. The set of reactions are summarized in Table 1, where *C*^*iα*^ is the molar concentration of species *i* in phase *α*, and *MW*_*i*_ is the molecular weight of species *i*.

Line 5 of Eq. (7) is a flux–force pair involving mass transfer between the fluid and solid phases. A conjugate flux–force closure for the mass transfer density rate is

(19) $$\overset{if\to is}{M}={\hat{K}}_{M}^{ifs}\left(\mu^{\bar{if}}+\psi^{\bar{f}}-\mu^{\bar{is}}-\psi^{\bar{s}}\right),$$

where ${\hat{K}}_{M}^{ifs}$ is a nonnegative mass-transfer rate coefficient.

Lines 6 and 7 of Eq. (7) are a flux–force pair involving momentum of the fluid phase. A conjugate flux–force closure can be formulated for the isotropic case as

(20) $$\nabla \left(\epsilon {\bar{\bar{f}}}_{p^{f}}\right)-\underset{i\in J_{s}}{\sum }\epsilon^{\bar{\bar{f}}}\rho^{f}\omega^{i\;\bar{f}}\left[\nabla \left(\mu^{\bar{if}}+\psi \bar{f}\right)+g^{\bar{f}}\right]+\overset{f\to fs}{T_{0}}={\hat{R}}^{f}\cdot \left(v^{\bar{f}}-v^{\bar{s}}\right),$$

where ${\hat{R}}^{f}$ is a positive definite resistance tensor for the fluid phase.

Line 8 of Eq. (7) involves the forces acting on the fluid–solid interface, and a conjugate flux–force closure can be written as

(21) $$-\nabla \cdot \left[\left(I-G^{fs}\right)\epsilon^{\bar{\bar{fs}}}\gamma^{fs}\right]-\overset{f\to fs}{T_{0}}-\overset{s\to fs}{T_{0}}={\hat{R}}^{fs}\cdot \left(v^{\bar{fs}}-v^{\bar{s}}\right),$$

where ${\hat{R}}^{fs}$ is a positive-semidefinite resistance tensor for the interface.

Line 9 of Eq. (7) involves the change in the porosity related to forces acting on the fluid–solid interface. A conjugate flux–force approximation can be written as

(22) $$\frac{{\text{D}}^{\bar{s}}\epsilon }{\text{D}t}=\hat{c}\left[p_{f}^{fs}+{\left(n_{s}\cdot t_{s}\cdot n_{s}\right)}_{s}^{fs}+\gamma^{fs}J_{s}^{fs}\right],$$

where $\hat{c}$ is a nonnegative solid compressibility coefficient.

The set of conjugate flux–force closure relations summarized in this subsection can be combined with a set of conservation of mass and momentum equations to yield a closed model. This model is formulated in the section that follows.

#### 6.2.3 Closed model

A closed two-phase model can be formulated by combining conservation equations given in Sect. 4 with closure relations given in Sect. 6.2.2. Because we are assuming isothermal conditions, the model will include conservation of species mass and entity momentum equations, but it will not be necessary to include conservation of energy equations. Other choices of secondary restrictions would result in different macroscale models, but the derivation process would follow a similar path.

The conservation of mass equations for a species in the fluid phase are

(23) $$\frac{{\text{D}}^{\bar{f}}\left(\epsilon \bar{\bar{f}}\rho^{f}\omega^{i\;\bar{f}}\right)}{\text{D}t}+\epsilon^{\bar{\bar{f}}}\rho^{f}\omega^{i\;\bar{f}}I:d^{\bar{\bar{f}}}+\nabla \cdot \left(\epsilon^{\bar{\bar{f}}}\rho^{f}\omega^{i\;\bar{f}}u^{\bar{\bar{if}}}\right)-\overset{is\to if}{M}=0\text{ for }i\in \left\{g,o,c,x\right\},$$

and for the solid phase

(24) $$\frac{{\text{D}}^{\bar{s}}\left(\epsilon^{\bar{\bar{s}}}\rho^{s}\omega^{i\;\bar{s}}\right)}{\text{D}t}+\epsilon^{\bar{\bar{s}}}\rho^{s}\omega^{i\;\bar{s}}I:d^{\bar{\bar{s}}}+\nabla \cdot \left(\epsilon^{\bar{\bar{s}}}\rho^{s}\omega^{i\;\bar{s}}u^{\bar{\bar{is}}}\right)-\epsilon^{\bar{\bar{s}}}r^{is}-\overset{if\to is}{M}=0\text{ for }i\in \left\{l,n,g,o,c,w,y\right\},$$

where the dominant species for each phase is eliminated using the constraint equation

(25) $$\underset{i\in J_{s}}{\sum }\omega^{i\;\bar{\alpha }}=1\text{ for }\alpha \in J_{\text{P}},$$

where $J_{\text{P}}$ is the index set of phases and some species are omitted from each phase by definition. Thus, four species conservation of mass equations exist for the fluid phase, and seven species conservation of mass equations exist for the solid phase. Combining these equations with constraints given by Eq. (25) fully specifies the composition of both phases.

Equations (23) and (24) contain terms involving deviation velocities, reactions, and interphase mass transfer. The previously derived closure relations can be used to explicitly specify these equations. Substituting Equations (14) and (19) into Eq. (23) yields the species conservation of mass equation for the fluid phase

(26) $$\frac{{\text{D}}^{\bar{f}}\left(\epsilon^{\bar{\bar{f}}}\rho^{f}\omega^{i\;\bar{f}}\right)}{\text{D}t}+\epsilon^{\bar{\bar{f}}}\rho^{f}\omega^{i\;\bar{f}}I:d^{\bar{\bar{f}}}-\nabla \cdot \left[\epsilon^{\bar{\bar{f}}}\rho^{f}{\hat{D}}_{iw}^{f}\left(\mu^{\bar{if}}-\mu^{\bar{wf}}\right)\right]+{\hat{K}}_{M}^{ifs}\left(\mu^{\bar{if}}+\psi^{\bar{f}}-\mu^{\bar{is}}-\psi^{\bar{s}}\right)=0\text{ for }i\in \left\{g,o,c,x\right\},$$

and for the solid phase

(27) $$\frac{{\text{D}}^{\bar{s}}\left(\epsilon^{\bar{\bar{s}}}\rho^{s}\omega^{i\;\bar{s}}\right)}{\text{D}t}+\epsilon^{\bar{\bar{s}}}\rho^{s}\omega^{i\;\bar{s}}I:d^{\bar{\bar{s}}}-\nabla \cdot \left[\epsilon^{\bar{\bar{f}}}\rho^{f}{\hat{D}}_{ie}^{s}\left(\mu^{\bar{is}}-\mu^{\bar{es}}\right)\right]\;+\epsilon^{\bar{\bar{s}}}\underset{k\in J_{\text{rxn}is}}{\sum }{\hat{K}}_{ks}v_{iks}MW_{i}\left(\underset{i\in J_{s}}{\sum }\mu^{\bar{is}}v_{iks}MW_{i}\right)\;-{\hat{K}}_{M}^{ifs}\left(\mu^{\bar{if}}+\psi^{\bar{f}}-\mu^{\bar{is}}-\psi^{\bar{s}}\right)=0\text{ for }i\in \left\{l,n,g,o,c,w,y\right\},$$

where only the reactions involving species *i* need to be considered for the *i* species transport equation, which is specified by the summation over index set $J_{\text{rxn}is}$ that is the set of all reactions in the *s* entity that involve species *i*.

Chemical potentials can be written in terms of mass fractions to minimize the closure problem with the conservation of mass equations. The macroscale chemical potential may be written as

(28) $$\mu^{\bar{i\alpha }}=\mu_{0}^{\bar{i\alpha }}\left(p^{\alpha },\theta^{\bar{\bar{\alpha }}}\right)+\frac{R_{g}\theta^{\bar{\bar{\alpha }}}}{MW_{i}}\text{ln}\left(x^{\bar{\bar{i\alpha }}}{\hat{\gamma }}^{\bar{i\alpha }}\right),$$

where $\mu_{0}^{\bar{i\alpha }}\left(p^{\alpha },\theta^{\bar{\bar{\alpha }}}\right)$ is a reference state chemical potential, *R*_*g*_ is the ideal gas constant, $x^{\bar{\bar{i\alpha }}}$ is a mole fraction, and ${\hat{\gamma }}^{\bar{\bar{i\alpha }}}$ is an activity coefficient, which we will assume is equal to 1 for all species and all compositions encountered. The mole fraction can be written as

(29) $$\omega^{i\;\bar{\alpha }}=\frac{MW_{i}}{MW_{\alpha }}x^{\bar{\bar{i\alpha }}},$$

where the molecular weight of the *α* entity is defined as

(30) $$MW_{\alpha }=\sum_{i\in J_{s}}MW_{i}x^{\bar{\bar{i\alpha }}}=\sum_{i\in J_{s}}{\left(\frac{\omega^{i\;\bar{\alpha }}}{MW_{i}}\right)}^{-1}.$$

Equation (1) can also be summed over all species, which yields an overall conservation of mass equation for an entity. This equation can be further expanded using an approximation for the macroscale velocity, which follows from the conservation of momentum equations that follow. These standard manipulations are detailed in the literature [19].

Recall the conservation of momentum equation, which can be written as

(31) $$\frac{{\text{D}}^{\bar{\alpha }}\left(\epsilon^{\bar{\bar{\alpha }}}\rho^{\alpha }v^{\bar{\alpha }}\right)}{\text{D}t}+\epsilon^{\bar{\bar{\alpha }}}\rho^{\alpha }v^{\bar{\alpha }}I:d^{\bar{\bar{\alpha }}}-\epsilon^{\bar{\bar{\alpha }}}\rho^{\alpha }g^{\bar{\alpha }}\;-\underset{\kappa \in J_{\text{c}\alpha }}{\sum }\underset{i\in J_{s}}{\sum }\overset{i\kappa \to i\alpha }{M}\left(v^{\bar{\bar{\alpha ,\kappa }}}+u_{i}^{\bar{\bar{\alpha ,\kappa }}}\right)-\underset{\kappa \in J_{\text{c}\alpha }}{\sum }\overset{\kappa \to \alpha }{T_{0}}\;-\nabla \cdot \left(\epsilon^{\bar{\bar{\alpha }}}t^{\bar{\bar{\alpha }}}\right)=0\text{ for }\alpha \in J.$$

This equation may be combined with the closure relations and manipulated to deduce a pair of closed momentum equations for the phases. The interface momentum equation is trivial because of the secondary restriction specifying a massless interface. Equations (9), (19), and (20) can be substituted into Eq. (31) to yield the resultant fluid phase momentum equation

(32) $$\underset{i\in J_{s}}{\sum }\epsilon^{\bar{\bar{f}}}\rho^{f}\omega^{i\;\bar{f}}\nabla \mu^{\bar{if}}-\epsilon^{\bar{\bar{f}}}\rho^{f}g^{\bar{f}}+{\hat{R}}^{f}\cdot \left(v^{\bar{f}}-v^{\bar{s}}\right)+\underset{i\in J_{s}}{\sum }{\hat{K}}_{M}^{ifs}\left(\mu^{\bar{if}}+\psi^{\bar{f}}-\mu^{\bar{is}}-\psi^{\bar{s}}\right)\left(v^{\bar{f}}+u^{\bar{\bar{if}}}\right)=0,$$

or approximated using the Gibbs–Duhem equation as [19]

(33) $$\epsilon^{\bar{\bar{f}}}\nabla p^{f}-\epsilon^{\bar{\bar{f}}}\rho^{f}g^{\bar{f}}+{\hat{R}}^{f}\cdot \left(v^{\bar{f}}-v^{\bar{s}}\right)+\underset{i\in J_{s}}{\sum }{\hat{K}}_{M}^{ifs}\left(\mu^{\bar{if}}+\psi^{\bar{f}}-\mu^{\bar{is}}-\psi^{\bar{s}}\right)\left(v^{\bar{f}}+u^{\bar{\bar{if}}}\right)=0,$$

where the inertial terms have been dropped due to the slow dynamics of the system and the area averaged velocities multiplying the mass exchange approximations have been replaced with the volume averages, as specified in secondary restrictions SR7-2P and SR8-2P.

Equation (31) can be summed over all entities, eliminating the momentum exchange terms, and yielding after substitution of the closure relations for the macroscale stress tensors

(34) $$\frac{{\text{D}}^{\bar{f}}\left(\epsilon^{\bar{\bar{f}}}\rho^{f}v^{\bar{f}}\right)}{\text{D}t}+\frac{{\text{D}}^{\bar{s}}\left(\epsilon^{\bar{\bar{s}}}\rho^{s}v^{\bar{s}}\right)}{\text{D}t}+\epsilon^{\bar{\bar{f}}}\rho^{f}v^{\bar{f}}I:d^{\bar{\bar{f}}}+\epsilon^{\bar{\bar{s}}}\rho^{s}v^{\bar{s}}I:d^{\bar{\bar{s}}}-\epsilon^{\bar{\bar{f}}}\rho^{f}g^{\bar{f}}-\epsilon^{\bar{\bar{s}}}\rho^{s}g^{\bar{s}}-\nabla \cdot \left[-\epsilon^{\bar{\bar{f}}}p^{f}I+\epsilon^{\bar{\bar{s}}}t^{s}+\gamma^{fs}\left(I-G^{fs}\right)\right]=0.$$

Further manipulations using this equation are possible, and expressions for the stress tensor have been developed using TCAT [20]. The end objective of such a momentum equation is to determine the velocity of the solid phase. Conditions may exist in which this velocity can be approximated without consideration of the details of solid mechanics. The existence of mass exchange complicates the usual solid mechanics mapping between Eulerian and Lagrangian coordinate systems, and open research questions remain, which will not be considered in this work.

## 7 Three-phase system

### 7.1 Description

For the two-phase system considered above, the tumor species were considered part of the solid phase. However, it is generally accepted that cells and tissues may be modeled as fluids [14]. Such approaches may be used to model adhesion of cells among themselves, to the ECM, and to substrates [1,30,56], and to model chemotaxis and haptotaxis to represent active cellular movement, such as observed in angiogenesis, invasion, and branching [9,31].

Hence, the purpose of this section is to consider a more complicated three-phase model involving tumor growth. This will require defining the entities, species in the entities, reactions, and other aspects of the model. An alternative form of the SEI will be relied upon to guide model closure. The basic model-building steps will parallel those steps followed to formulate the two-phase model presented in the previous section.

The three phases consist of a solid phase, *s*, a wetting fluid denoted *w*, and a non-wetting fluid phase denoted *n*. The wetting fluid preferentially wets the solid phase compared to the non-wetting fluid phase, although a contact angle can exist between the fluid–fluid interface and the solid surface; measured through the wetting fluid phase this contact angle will be < 90 degrees and 0 degrees for a strongly water wet solid phase. The index set of all entities is

(35) J={w,n,s,wn,ws,ns,wns},

where the grouping of two symbols denotes the interface that forms between the two respective phases and *wns* is an index for the common curve formed where three phases meet.

The index set of the ten species considered is identical to the set previously considered for the two-phase tumor model with the exception that an additional collective background species *z* is added to the set

(36) $$J_{s}=\left\{l,n,e,g,o,c,w,x,y,z\right\}.$$

Table 2 summarizes the entities, the index used for each entity, and species composition of each entity in the system; the composition of interfaces and common curves will not be resolved in this restricted model, so the composition of these entities is neglected. The primary restrictions for the three-phase model are identical to the primary restrictions for the previously presented two-phase model. The secondary restrictions are similar to the secondary restrictions for the two-phase model, although not identical: (SR1-3P) the system is isothermal; (SR2-3P) the interfaces and common curves are massless, (SR3-3P) kinetic energy terms are higher order and of negligible importance; (SR4-3P) body force vectors and potentials are identical for all species; (SR5-3P) chemical reactions can be formulated in terms of chemical affinities; (SR6-3P) inertial terms in the momentum equations are insignificant due to the slow dynamics of the systems considered; (SR7-3P) density-weighted, area-averaged velocities and deviation velocities are equal to their volume-averaged counterparts; (SR8-3P) the Lagrangian stress tensor product with the Green’s deformation tensor can be neglected from the driving force difference for mass transfer to the solid phase; (SR9-3P) the activity coefficient of all species is unity; (SR10-3P) common curve effects are considered higher order and neglected; (SR11-3P) the contact angle is considered constant as a result of the relatively slow dynamics of tumor growth; and (SR12-3P) the curvature of the solid phase is not dependent upon the fluid wetting the solid surface.

### 7.2 Formulation

The three-phase model described above is formulated into a closed model following a similar approach that was taken for the simpler two-phase model formulated above. The sections that follow detail the restricted SEI, the closure relations, and the closed model.

#### 7.2.1 Restricted SEI

Applying the secondary restrictions specified in Sect. 7.1 to the general SEI given by Eq. (4) results in the restricted SEI for a compositional three-phase system given by

(37) $$\sum_{\alpha \in J_{\text{f}}}\frac{1}{\theta }\left(\epsilon^{\bar{\bar{\alpha }}}t^{\bar{\bar{\alpha }}}+\epsilon^{\bar{\bar{\alpha }}}p^{\alpha }I\right):d^{\bar{\bar{\alpha }}}+\frac{1}{\theta }\left(\epsilon^{\bar{\bar{s}}}{\bar{t}}^{\bar{\bar{s}}}-\epsilon^{\bar{\bar{s}}}t^{s}\right):{\text{d}}^{\bar{\bar{s}}}\;+\sum_{\alpha \in J_{I}}\frac{1}{\theta }\left[\epsilon^{\bar{\bar{\alpha }}}t^{\bar{\bar{\alpha }}}-\epsilon^{\bar{\bar{\alpha }}}\gamma^{\alpha }\left(I-G^{\alpha }\right)\right]:d^{\bar{\bar{\alpha }}}\;-\sum_{\alpha \in J_{\text{P}}}\sum_{i\in J_{s}\backslash N}\frac{1}{\theta }\epsilon^{\bar{\bar{\alpha }}}\rho^{\alpha }\omega^{i\;\bar{\alpha }}u^{\bar{\bar{i\alpha }}}\cdot \nabla \left(\mu^{\bar{i\alpha }}-\mu^{\bar{N\alpha }}\right)\;-\sum_{\alpha \in J_{\text{P}}}\sum_{k\in J_{\text{rxn}\alpha }}\frac{1}{\theta }\epsilon^{\bar{\bar{\alpha }}}R^{k\alpha }A^{k\alpha }\;+\sum_{i\in J_{s}}\overset{iw\to in}{M}\frac{1}{\theta }\left(\mu^{\bar{iw}}+\psi^{\bar{w}}-\mu^{\bar{in}}-\psi^{\bar{n}}\right)\;+\sum_{i\in J_{s}}\overset{iw\to is}{M}\frac{1}{\theta }\left(\mu^{\bar{iw}}+\psi^{\bar{w}}-\mu^{\bar{is}}-\psi^{\bar{s}}\right)\;+\sum_{i\in J_{s}}\overset{in\to is}{M}\frac{1}{\theta }\left(\mu^{\bar{in}}+\psi^{\bar{n}}-\mu^{\bar{is}}-\psi^{\bar{s}}\right)\;-\frac{1}{\theta }\{-\nabla \left(\epsilon^{\bar{\bar{w}}}p^{w}\right)+\epsilon^{\bar{\bar{w}}}\rho^{w}\left(\sum_{i\in J_{s}}\omega^{i\;\bar{w}}\nabla \mu^{\bar{iw}}+\nabla \psi^{\bar{w}}+g^{\bar{w}}\right)\;-\overset{w\to wn}{T_{0}}-\overset{w\to ws}{T_{0}}\}\cdot \left(v^{\bar{w}}-|v^{\bar{s}}\right)\;-\frac{1}{\theta }\{-\nabla \left(\epsilon^{\bar{\bar{n}}}p^{n}\right)+\epsilon^{\bar{\bar{n}}}\rho^{n}\left(\sum_{i\in J_{s}}\omega^{i\;\bar{n}}\nabla \mu^{\bar{in}}+\nabla \psi^{\bar{n}}+g^{\bar{n}}\right)\;-\overset{n\to wn}{T_{0}}-\overset{n\to ns}{T_{0}}\}\cdot \left(v^{\bar{n}}-v^{\bar{s}}\right)\;-\sum_{\alpha \in J_{\text{I}}}\frac{1}{\theta }\left\{\nabla \cdot \left[\left(I-G^{\alpha }\right)\epsilon^{\bar{\bar{\alpha }}}\gamma^{\alpha }\right]+\sum_{\kappa \in J_{c\alpha }^{+}}\overset{\kappa \to \alpha }{T_{0}}\right\}\cdot \left(v^{\bar{\alpha }}-v^{\bar{s}}\right)\;+\frac{1}{\theta }\left[\frac{{\text{D}}^{\bar{s}}\epsilon^{\bar{\bar{w}}}}{\text{D}t}-\chi_{s}^{\bar{\bar{ws}}}\frac{{\text{D}}^{\bar{s}}\epsilon }{\text{D}t}-\frac{\gamma^{wn}{\hat{k}}_{1}^{wn}\left(\epsilon^{\bar{\bar{\omega n}}}-\epsilon_{\text{eq}}^{\bar{\bar{wn}}}\right)}{p_{w}^{wn}-p_{n}^{wn}}\right]\;\times \left(p_{w}^{wn}-p_{n}^{wn}-\gamma^{wn}J_{w}^{wn}\right)\;+\frac{1}{\theta }\chi_{s}^{\bar{\bar{ws}}}\frac{{\text{D}}^{\bar{s}}\epsilon }{\text{D}t}\left[p_{w}^{ws}+{\left(n_{s}\cdot t_{s}\cdot n_{s}\right)}_{s}^{ws}+\gamma^{ws}J_{s}^{ws}\right]\;+\frac{1}{\theta }\chi_{s}^{\bar{\bar{ns}}}\frac{{\text{D}}^{\bar{s}}\epsilon }{\text{D}t}\left[p_{n}^{ns}+{\left(n_{s}\cdot t_{s}\cdot n_{s}\right)}_{s}^{ns}+\gamma^{ns}J_{s}^{ns}\right]\;=\sum_{\alpha \in J}\Lambda^{\bar{\bar{\alpha }}}\geq 0.$$

Equation (37) can be used to generate closure relations that are consistent with the second law of thermodynamics. While the form of this equation is much simpler than the general case given by Eq. (4) because of the secondary restrictions applied, it is significantly more complicated than the two-phase SEI given by Eq. (7) as a result of the additional entities that must be considered.

#### 7.2.2 Closure relations

The procedure suggested for deriving closure relations is similar to that used for the two-phase case, although additional relations are needed for the three-phase case. We consider the first 16 lines of Eq. (37) to generate a candidate set of closure relations, which will in turn be used to formulate a closed model in the following section.

A zero-order closure for the fluid phases yields from Line 1 of Eq. (37)

(38) $$t^{\bar{\bar{\alpha }}}=-p^{\alpha }I\text{ for }\alpha \in J_{\text{f}},$$

and for the solid phase

(39) $$t^{\bar{\bar{s}}}=t^{s}.$$

A zero-order approximation based on Line 2 yields an approximation for the stress tensor for the interfaces

(40) $$t^{\bar{\bar{\alpha }}}=\gamma^{\alpha }\left(I-G^{\alpha }\right)\text{ for }\alpha \in J_{\text{I}}.$$

A first-order conjugate flux–force approximation based upon Line 3 in Eq. (37) yields the following approximations for the deviation velocities in each of the phases

(41) $$\omega^{i\;\bar{w}}u^{\bar{\bar{iw}}}=-{\hat{D}}_{iw}^{w}\cdot \nabla \left(\mu^{\bar{iw}}-\mu^{\bar{ww}}\right)\text{ for }i\in \left\{g,o,c,z\right\},$$

(42) $$\omega^{i\;\bar{n}}u^{\bar{\bar{in}}}=-{\hat{D}}_{il}^{n}\cdot \nabla \left(\mu^{\bar{in}}-\mu^{\bar{ln}}\right)\text{ for }i\in \left\{n,g,o,c,w,x\right\},$$

and

(43) $$\omega^{i\;\bar{s}}u^{\bar{\bar{is}}}=-{\hat{D}}_{ie}^{s}\cdot \nabla \left(\mu^{\bar{is}}-\mu^{\bar{es}}\right)\text{ for }i\in \left\{g,o,w,y\right\},$$

where water is taken as the reference species in the wetting phase, the live tumor species is the reference species in the non-wetting phase, and the extra-cellular matrix species is the reference species for the solid phase.

Line 4 in Eq. (37) can be used in a conjugate flux–force form to approximate thermodynamically consistent reaction rates. The manner in which this is done is analogous to the approach previously detailed for the simpler two-phase model. An example set of reactions are detailed in Table 3. The reactions are related to tumor growth, destruction, necrotic species formation, and lysis. All reactions occur in the non-wetting fluid phase. We emphasize that this is merely an example set of reactions and alternative reaction sets are permissible. The general expression for reactions in phases is

(44) $$r^{i\alpha }=-\underset{k\in J_{\text{rxn}\alpha }}{\sum }{\hat{K}}_{k\alpha }v_{ik\alpha }MW_{i}\left(\underset{i\in J_{s}}{\sum }\mu^{\bar{i\alpha }}v_{ik\alpha }MW_{i}\right)\text{ for }\alpha \in J_{\text{P}}.$$

A first-order conjugate form closure relation for the rate of mass transfer can be deduced from Lines 5–7 in Eq. (37) and is of the form

(45) $$\overset{i\alpha \to i\beta }{M}={\hat{K}}_{M}^{i\alpha \beta }\left(\mu^{\bar{i\alpha }}+\psi^{\bar{\alpha }}-\mu^{\bar{i\beta }}-\psi^{\bar{\beta }}\right)\text{ for }\alpha ,\beta \in J_{\text{P}},$$

where the indices denote that mass transfer can occur between any of three binary combinations of phases.

Lines 8–11 of Eq. (37) can be used to generate cross-coupled approximations involving the interphase transfer of momentum for the fluid phases of the form

(46) $$\nabla \left(\epsilon^{\bar{\bar{w}}}p^{w}\right)-\underset{i\in J_{s}}{\sum }\epsilon^{\bar{\bar{w}}}\rho^{w}\omega^{i\;\bar{w}}\left[\nabla \left(\mu^{\bar{iw}}+\psi^{\bar{w}}\right)+g^{\bar{w}}\right]+\overset{w\to wn}{T_{0}}+\overset{w\to ws}{T_{0}}=\underset{\kappa \in J_{\text{f}}}{\sum }{\hat{R}}_{\kappa }^{w}\cdot \left(v^{\bar{\kappa }}-v^{\bar{s}}\right),$$

and

(47) $$\nabla \left(\epsilon^{\bar{\bar{n}}}p^{n}\right)-\underset{i\in J_{s}}{\sum }\epsilon^{\bar{\bar{n}}}\rho^{n}\omega^{i\;\bar{n}}\left[\nabla \left(\mu^{\bar{in}}+\psi^{\bar{n}}\right)+{\text{g}}^{\bar{n}}\right]+\overset{n\to wn}{T_{0}}+\overset{n\to ns}{T_{0}}=\underset{\kappa \in J_{\text{f}}}{\sum }{\hat{R}}_{\kappa }^{n}\cdot \left(v^{\bar{\kappa }}-v^{\bar{s}}\right),$$

where $\hat{R}$ is a positive semi-definite resistance tensor.

A first-order approximation based upon Line 12 in Eq. (37) yields (47)

(48) $$\nabla \cdot \left[\left(I-G^{\alpha }\right)\epsilon^{\bar{\bar{\alpha }}}\gamma^{\alpha }\right]+\underset{\kappa \in J_{\text{c}\alpha }^{+}}{\sum }\overset{\kappa \to \alpha }{T_{0}}=-{\hat{R}}_{\alpha }^{\alpha }\cdot \left(v^{\bar{\alpha }}-v^{\bar{s}}\right)-\underset{\kappa \in J_{\text{c}\alpha }^{+}}{\sum }{\hat{R}}_{\kappa }^{\alpha }\cdot \left(v^{\bar{\kappa }}-v^{\bar{s}}\right)\text{ for }\alpha \in J_{\text{I}}.$$

Lines 13 and 14 from Eq. (37) can be used to develop a first-order approximation for the relaxation of capillary pressure to its equilibrium state, which is the case when the term in Line 14 vanishes. The resulting approximation is

(49) $$\frac{{\text{D}}^{\bar{s}}\epsilon^{\bar{\bar{w}}}}{\text{D}t}-\chi_{s}^{\bar{\bar{ws}}}\frac{{\text{D}}^{\bar{s}}\epsilon }{\text{D}t}-\frac{\gamma^{wn}{\hat{k}}_{1}^{wn}\left(\epsilon^{\bar{\bar{wn}}}-\epsilon_{\text{eq}}^{\bar{\bar{wn}}}\right)}{p_{w}^{wn}-p_{n}^{wn}}={\hat{c}}^{wn}\left(p_{w}^{wn}-p_{n}^{wn}-\gamma^{wn}J_{w}^{wn}\right),$$

where ${\hat{c}}^{wn}$ is a positive capillary pressure relaxation coefficient.

As a result of SR12-3P, the curvature of the solid phase simplifies to

(50) $$J_{s}^{ss}=J_{s}^{ws}=J_{s}^{ns},$$

which provide an approximation for the change in porosity as a function of normal forces on the solid surface of the form

(51) $$\frac{{\text{D}}^{\bar{s}}\epsilon }{\text{D}t}={\hat{c}}^{ss}\left[\chi_{s}^{\bar{\bar{ws}}}p_{w}^{wn}+\chi_{s}^{\bar{ns}}p_{n}^{ns}+{\left(n_{s}\cdot t_{s}\cdot n_{s}\right)}^{ss}+\left(\chi_{s}^{\bar{\bar{ws}}}\gamma^{ws}+\chi_{s}^{\bar{\bar{ns}}}\gamma^{ns}\right)J_{s}^{ss}\right],$$

where ${\hat{c}}^{ss}$ is a positive compressibility coefficient.

In addition to the closure relations that can be deduced from the SEI, evolution equations based upon averaging theorems [19,22] can also be used to close the three-phase model. The use of such evolution equations to produce closed models is an important aspect of TCAT models. For the three-phase model considered here, an evolution equation for the fluid–fluid interfacial area can be written as

(52) $$\frac{{\text{D}}^{\bar{s}}\epsilon^{\bar{\bar{wn}}}}{\text{D}t}+\nabla \cdot \left[\epsilon^{\bar{\bar{wn}}}\left(w^{wn}-G^{wn}\cdot v^{\bar{s}}\right)\right]+\epsilon^{\bar{\bar{wn}}}G^{wn}:d^{\bar{\bar{s}}}-J_{w}^{wn}\left(\frac{{\text{D}}^{\bar{s}}\epsilon^{\bar{\bar{w}}}}{\text{D}t}+\chi_{s}^{\bar{\bar{ws}}}\frac{{\text{D}}^{\bar{s}}\epsilon^{\bar{\bar{s}}}}{\text{D}t}\right)\;-{\hat{k}}^{wn}\left(\epsilon_{\text{eq}}^{\bar{\bar{wn}}}-\epsilon^{\bar{\bar{wn}}}\right)-\text{cos }\varphi^{\bar{\bar{ws,wn}}}\left(\epsilon^{\bar{\bar{ws}}}+\epsilon^{\bar{\bar{ns}}}\right)\frac{{\text{D}}^{\bar{s}}\chi_{s}^{\bar{\bar{ws}}}}{\text{D}t}\;+\text{sin }\varphi^{\bar{\bar{ws,wn}}}\frac{\epsilon^{\bar{\bar{wns}}}}{\epsilon^{\bar{\bar{ws}}}+\epsilon^{\bar{\bar{ns}}}}\frac{{\text{D}}^{\bar{s}}\epsilon^{\bar{\bar{s}}}}{\text{D}t}=0,$$

where neglecting common curve contributions this equation simplifies to

(53) $$\frac{{\text{D}}^{\bar{s}}\epsilon^{\bar{\bar{wn}}}}{\text{D}t}+\nabla \cdot \left[\epsilon^{\bar{\bar{wn}}}\left(w^{wn}-G^{wn}\cdot v^{\bar{s}}\right)\right]+\epsilon^{\bar{\bar{wn}}}G^{wn}:d^{\bar{\bar{s}}}-J_{w}^{wn}\left(\frac{{\text{D}}^{\bar{s}}\epsilon^{\bar{\bar{w}}}}{\text{D}t}+\chi_{s}^{\bar{\bar{ws}}}\frac{{\text{D}}^{\bar{s}}\epsilon^{\bar{\bar{s}}}}{\text{D}t}\right)-{\hat{k}}^{wn}\left(\epsilon_{\text{eq}}^{\bar{\bar{wn}}}-\epsilon^{\bar{\bar{wn}}}\right)-\text{cos }\varphi^{\bar{\bar{ws,wn}}}\left(\epsilon^{\bar{\bar{ws}}}+\epsilon^{\bar{\bar{ns}}}\right)\frac{{\text{D}}^{\bar{s}}\chi_{s}^{\bar{\bar{ws}}}}{\text{D}t}=0.$$

**G**^*wn*^ is a diagonal tensor of trace 1 for the isotropic case, which is a reasonable approximation. For other cases, an evolution equation for this quantity is needed, which can be derived at the microscale, averaged to the macroscale, and approximated in a convenient form.

The interfacial velocity vector is given by **w**^*wn*^, which is the macroscale velocity of the fluid–fluid interface, which moves in a direction normal to the interface. An exact equation for this kinematic quantity is not available.

An approximation can be derived based upon the averaging theorems of the form

(54) $$\epsilon^{{\bar{\bar{wn}}}^{2}}w^{wn}=-\frac{\partial \epsilon^{\bar{\bar{w}}}}{\partial t}\left(\nabla \epsilon^{\bar{\bar{w}}}+{\langle n_{w}\rangle }_{\Omega_{ws},\Omega }\right),$$

where an approximation is needed for the average of the normal vector, which is trivial for the common isotropic case.

As surfaces deform, their curvatures change. An evolution equation relating the change in the mean and the Gaussian curvature can be written as

(55) $$\epsilon^{\bar{\bar{wn}}}\frac{\partial K_{n}^{wn}}{\partial t}+K_{n}^{wn}\frac{\partial \epsilon^{\bar{\bar{wn}}}}{\partial t}+\nabla \cdot \left(\epsilon^{\bar{\bar{\omega n}}}K_{n}^{wn}w^{wn}\right)\;+\frac{J_{n}^{wn}}{2}\nabla \nabla :\left[\left(I-G^{wn}\right)\frac{\partial \epsilon^{\bar{\bar{n}}}}{\partial t}\right]\;+\frac{J_{n}^{wn}}{2}\nabla \cdot \left(\epsilon^{\bar{\bar{wn}}}J_{n}^{wn}w^{wn}\right)=0.$$

A constraint equation based upon Galilean invariance for the mean curvature is

(56) $$-\nabla \cdot \left[\epsilon^{\bar{\bar{wn}}}J_{n}^{wn}\left(I-2G^{wn}\right)\right]+2K_{n}^{wn}\nabla \epsilon^{\bar{n}}-\nabla \nabla :\left(\left(I-G^{wn}\right)\nabla \epsilon^{\bar{\bar{n}}}\right)=0,$$

and the Gaussian curvatures can be related to the Euler characteristic for smooth closed boundaries using the Gauss Bonnet theorem giving

(57) $$4\pi \chi^{\bar{\bar{n}}}=\epsilon^{\bar{\bar{wn}}}K_{n}^{wn}+\epsilon^{\bar{\bar{ns}}}K_{n}^{ns}.$$

Additional closure relations for the three-phase model are available through the specification of state equations. Another example of a state equation is a formulation that can be deduced from integral geometry that relates volume fractions, interfacial areas, and curvatures of the form [47]

(58) $$\epsilon^{\bar{\bar{n}}}=F\left(\epsilon^{\bar{\bar{wn}}}+\epsilon^{\bar{\bar{ns}}},\epsilon^{\bar{\bar{wn}}}J_{w}^{wn}+\epsilon^{\bar{\bar{ns}}}J_{s}^{ns},\chi^{\bar{\bar{n}}}\right),$$

where *F* is a smooth differentiable function that can be deduced from state data. Equation (58) has been shown to provide an accurate representation of capillary pressure that is based on theory, hysteretic free—unlike traditional capillary pressure equations, and applicable for not only equilibrium conditions but also dynamic states as well [23]. Equations of state can be formulated to relate densities to pressures and compositions as well.

#### 7.2.3 Closed model

The purpose of this section is to assemble the pieces needed for a complete, closed three-phase model of tumor growth, which can be used as a basis to derive numerical approximations. The three-phase model is more complicated than the previously formulated two-phase model. This results from the existence of additional entities and the attendant closure problem. Overall conservation equations for mass and momentum are needed for each phase, and compositional equations including mass transfer and reactions are needed for the species in a phase. The massless interface and common curve assumptions do simplify the formulation for lower dimensional entities. We note that other TCAT formulation approaches are possible, and we are merely providing an example for completeness. This model follows from the conservation equations and closure relations based upon the SEI that were previously presented. The quantities we wish to resolve include the composition of each phase, the velocity and mass density of each phase, fluid pressures and solid stresses, entity extents, and interfacial curvatures needed to approximate the state of the system. The model that follows is based upon these considerations.

Summing Eq. (1) over all species yields a set of conservation of mass equations for the phases of the form

(59) $$\frac{{\text{D}}^{\bar{\alpha }}\left(\epsilon^{\bar{\bar{\alpha }}}\rho^{\alpha }\right)}{\text{D}t}+\epsilon^{\bar{\bar{\alpha }}}\rho^{\alpha }I:d^{\bar{\bar{\alpha }}}-\underset{i\in J_{s}}{\sum }\underset{\kappa \in J_{\text{c}\alpha }}{\sum }\overset{i\kappa \to i\alpha }{M}=0\text{ for }\alpha \in J_{\text{P}},$$

which can be written using Eq. (45) as

(60) $$\frac{{\text{D}}^{\bar{\alpha }}\left(\epsilon^{\bar{\bar{\alpha }}}\rho^{\alpha }\right)}{\text{D}t}+\epsilon^{\bar{\bar{\alpha }}}\rho^{\alpha }I:d^{\bar{\bar{\alpha }}}-\underset{i\in J_{s}}{\sum }\underset{\kappa \in J_{\text{c}\alpha }}{\sum }{\hat{K}}_{M}^{i\kappa \alpha }\left(\mu^{\bar{i\kappa }}+\psi^{\bar{\kappa }}-\mu^{\bar{i\alpha }}-\psi^{\bar{\alpha }}\right)=0\text{ for }\alpha \in J_{\text{P}},$$

where the chemical potentials can be written in terms of mole fractions and mass fractions as previously formulated in Eqs. (28) and (30).

Species composition for each phase follows from

(61) $$\frac{{\text{D}}^{\bar{\alpha }}\left(\epsilon^{\bar{\bar{\alpha }}}\rho^{\alpha }\omega^{i\;\bar{\alpha }}\right)}{\text{D}t}+\epsilon^{\bar{\bar{\alpha }}}\rho^{\alpha }\omega^{i\;\bar{\alpha }}I:d^{\bar{\bar{\alpha }}}+\nabla \cdot \left(\epsilon^{\bar{\bar{\alpha }}}\rho^{\alpha }\omega^{i\;\bar{\alpha }}u^{\bar{\bar{i\alpha }}}\right)-\epsilon^{\bar{\bar{\alpha }}}r^{i\alpha }-\underset{\kappa \in J_{\text{c}\alpha }}{\sum }\overset{i\kappa \to i\alpha }{M}=0\text{ for }i\in J_{s},\;\alpha \in J_{\text{P}},$$

where the deviation velocities follow from substitution of Eqs. (41)–(43), reaction terms from Eq. (44), and mass transfer from Eq. (45). Note that a complete set of mass conservation equations must be formulated and solved subject to the constraint

(62) $$\underset{i\in J_{s}}{\sum }\omega^{i\;\bar{\alpha }}=1\text{ for }\alpha \in J_{\text{P}}.$$

Conservation of mass equations are not required for interfaces or the common curve because they have been specified as massless.

As a result of secondary restrictions SR6-3P and SR7-3P, Eq. (2) can be simplified, Eqs. (38), (45), and (46) substituted in and the resulting conservation of momentum equations for the fluid phases written as

(63) $$\underset{i\in J_{s}}{\sum }\epsilon^{\bar{\bar{\alpha }}}\rho^{\alpha }\omega^{i\;\bar{\alpha }}\nabla \mu^{\bar{i\alpha }}-\epsilon^{\bar{\bar{\alpha }}}\rho^{\alpha }g^{\bar{\alpha }}+\underset{\kappa \in J_{\text{f}}}{\sum }{\hat{R}}_{\kappa }^{\alpha }\cdot \left(v^{\bar{\kappa }}-v^{\bar{s}}\right)+\underset{i\in J_{s}}{\sum }\underset{\kappa \in J_{\text{p}}}{\sum }{\hat{K}}_{M}^{i\alpha \kappa }\left(\mu^{\bar{i\alpha }}+\psi^{\bar{\alpha }}-\mu^{\bar{i\kappa }}-\psi^{\bar{\kappa }}\right)\left(v^{\bar{\alpha }}+u^{\bar{\bar{i\alpha }}}\right)=0,$$

which can be approximated using the Gibbs–Duhem equation as [19]

(64) $$\epsilon^{\bar{\bar{\alpha }}}\nabla p^{\alpha }-\epsilon^{\bar{\bar{\alpha }}}\rho^{\alpha }g^{\bar{\alpha }}+\underset{\kappa \in J_{\text{f}}}{\sum }{\hat{R}}_{\kappa }^{\alpha }\cdot \left(v^{\bar{\kappa }}-v^{\bar{s}}\right)+\underset{i\in J_{s}}{\sum }\underset{\kappa \in J_{\text{p}}}{\sum }{\hat{K}}_{M}^{i\alpha \kappa }\left(\mu^{\bar{i\alpha }}+\psi^{\bar{\alpha }}-\mu^{\bar{i\kappa }}-\psi^{\bar{\kappa }}\right)\left(v^{\bar{\alpha }}+u^{\bar{\bar{i\alpha }}}\right)=0,$$

where viscous coupling of momentum transport between the fluid phases follows naturally from the cross-coupled closure relations [36].

A momentum equation is also required for the solid phase, which can alternatively be expressed as the total change in momentum for the system, which can be formulated summing Eq. (2) over all entities using the closure approximations given by Eqs. (38) and (40) yielding

(65) $$\frac{{\text{D}}^{\bar{w}}\left(\epsilon^{\bar{\bar{w}}}\rho^{w}v^{\bar{w}}\right)}{\text{D}t}+\frac{{\text{D}}^{\bar{n}}\left(\epsilon^{\bar{\bar{n}}}\rho^{n}v^{\bar{n}}\right)}{\text{D}t}+\frac{{\text{D}}^{\bar{s}}\left(\epsilon^{\bar{\bar{s}}}\rho^{s}v^{\bar{s}}\right)}{\text{D}t}+\epsilon^{\bar{\bar{w}}}\rho^{w}v^{\bar{w}}I:d^{\bar{\bar{w}}}+\epsilon^{\bar{\bar{n}}}\rho^{n}v^{\bar{n}}I:d^{\bar{\bar{n}}}\;+\epsilon^{\bar{\bar{s}}}\rho^{s}v^{\bar{s}}I:d^{\bar{\bar{s}}}-\epsilon^{\bar{\bar{w}}}\rho^{w}g^{\bar{w}}-\epsilon^{\bar{\bar{n}}}\rho^{n}g^{\bar{n}}-\epsilon^{\bar{\bar{s}}}\rho^{s}g^{\bar{s}}\;-\nabla \cdot \left[-\epsilon^{\bar{\bar{w}}}p^{w}I-\epsilon^{\bar{\bar{n}}}p^{n}I+\epsilon^{\bar{\bar{s}}}t^{s}+\sum_{\kappa \in J_{\text{I}}}\gamma^{\kappa }\left(I-G^{\kappa }\right)\right]=0.$$

In the event that a zero velocity at the macroscale is not a reasonable assumption for the solid phase, expressions available in the literature [20] can be used to produce a solvable equation. As previously noted, additional work on the solid mechanics of these biomechanical systems is warranted, and this is an active area of research[6,11].

The three-phase model is complete when augmented with evolution equations for the volume fraction given by Eq. (49) and the interfacial area given by Eq. (53), and equations of state given by Eq. (58) and state equations for the dependency of phase densities as a function of pressures and compositions. Approximations would also be needed for the specific interfacial area $\epsilon^{\bar{\bar{ns}}}$ and the mean curvature of the solid phase $J_{s}^{ns}$.

## 8 Discussion

Tumor occurrence and growth exhibits classical problems of scale. A complete understanding requires molecular, genetic, and cellular perspectives, while the systems of concern are at scales of the human body and thus several orders of magnitude above the length scales of fundamental concern. The perspective advanced herein is a practical approach focused on advancing a mechanistic mathematical description that is able to describe the system of concern. The TCAT approach provides a means to formulate such models, which are founded upon conservation principles, thermodynamics, and a set of mathematical theorems. Even though molecular and cellular approaches are essential to advance fundamental understanding of tumor formation and growth, the laws of continuum mechanics must still apply provided the necessary primary restrictions have been met. The approach taken is to develop macroscale models in which tumors are described in an averaged sense with admissible changes in both space and time. With this general approach, a wide variety of specific models is possible.

The primary goal of this work was to illustrate how the TCAT approach can be used to formulate macroscale models of varying sophistication and complexity. Two specific examples were provided: a two-phase formulation, and three-phase formulation; the former included a single fluid phase and the latter included two fluid phases and both included a solid phase. Both of these examples included interfaces, and the full three-phase case can include a common curve, which was included in the SEI but dropped for simplicity in the final example formulation. Both approaches include several species, reactions for tumor formation, tumor destruction, necrotic tissue formation, and necrotic lysis, and mass transfer. We emphasize that the examples provided are intended to be a starting point for advancement in understanding of the how the TCAT approach can be applied, evaluated, and validated. It is expected that details of the methods, especially the species and reaction sets, will evolve as understanding of the essential components of an optimally useful model also evolve. As understanding, and available computing power, expands, so too will the complexity and fidelity of the underlying models. However, the approaches illustrated will provide a solid foundation for continuum mechanical modeling of tumor growth.

Some previous tumor growth models have included elements of TCAT but not to the extent detailed in this work [32,53–55,60]. They have been successful in modeling tumor growth but not entirely satisfactory. Especially the missing interfaces required questionable approximations when linking capillary pressures with saturations. It is hoped that the presented approach will allow a step forward in this direction. The next steps beyond the numerical implementation of the detailed equations will be the inclusion of angiogenesis in the model and finally the addition of drug delivery. The number of parameters involved will require a sensitivity analysis to identify the leading order effects and to motivate the most important experimental work. Uncertainty quantification is strongly linked to this problem. It is hoped that mechanistic models of the sort presented herein will help to elucidate mechanisms resulting in cancer growth and treatment, which may be considered independent of the genetic origins. In fact, recent studies have revealed extensive variations in genetic signatures, gene expression, and post-translational modifications among different tumors and within tumors, creating complex tumor heterogeneities. Heterogeneity is frequently attributed to genetics, but it is related to non-genetic influences too [5]. Clearly, this directly impacts therapeutic treatments and outcomes [40,43,59]. Thus, better understanding of heterogeneities will help to improve treatments of metastatic diseases. This brings us to the final aim of our modeling effort which is the evaluation of the efficiency of cancer drugs. This needs an efficient model of tumor growth linked to a bio-distribution model of the drug under scrutiny.

A motivation for three-fluid-phase models would be a need to represent tumor systems based upon three distinct phases with immiscible fluid-like properties that form separate regions of matter with distinct interfacial tensions. While such models may be needed, much can be done with the general framework for one-fluid and two-fluid models advanced in this work. In the event that model evaluation and validation efforts of the sorts of models advanced in this work result in a conclusion that three-fluid-phase continuum models are needed to represent tumor growth with adequate fidelity, TCAT can be used to derive such models. While the conservation and balance equations are in place for such advancements, additional thermodynamic, evolution equation, and state equation work would be required. A new general SEI would also need to be derived. Of course, such a theoretical framework would be applicable to others systems as well.

Models with some similarities to those detailed herein are being used for successful medical applications (e.g. [6,38]), and extensions to clinical applications of drug delivery and evaluation of drug efficiency are also possible; initial works in these directions can be found in [4,10].

Because the intent of this work was to provide a foundational example of how TCAT can be used to formulate macroscale models to describe tumor growth, it has not included the numerical methods aspects of approximating these models. Furthermore, the worth of a model is dependent upon its ability to represent physical systems of concern with sufficient fidelity to be of use to those assessing such systems. Such an assessment requires not only an approximation of the formulation butal so parameter estimation and comparison to observed systems. It is hoped that this work will enable efforts to approximate, apply, evaluate, and validate a range of candidate TCAT models in pursuit of realistic and worthwhile representations of systems important to society. Much work remains to be done to fulfill this vision.

## 9 Conclusions

Several conclusions follow from this work:

1. Macroscale continuum mechanical approaches provide a means to describe tumor formation, growth, and various treatment modalities at a length scale relevant to human systems of concern.
2. TCAT provides a framework for formulating such macroscale models in a manner that is consistent across length scales with respect to established conservation and thermodynamic principles.
3. Two-phase and three-phase TCAT models were formulated as examples of the formulation process and reduced to a closed form.
4. The work advances prior efforts in providing a more complete set of entities and including recent advances in differential geometry evolution equations and integral geometry state equations, which provide accurate closure approximation for two-fluid porous medium models.
5. The foundational nature of this work is intended to support future work to improve upon the example formulations provided in this work in concert with numerical approximation of the formulated models, parameter estimation, evaluation, and validation.
6. It is anticipated that advances in fundamental molecular and cellular level understanding of tumor formation, growth, and treatment processes will inform and help improve continuum scale models of focus in this work, which may enable more routine simulation at scales of concern and help advance more effective treatment approaches.

## Figures and Tables

**Table 1:** Reactions for two-phase systems

Description	Reaction	References
Tumor formation	*ν*_*gfs*_ *MW*_*g*_*C*^*gs*^ + *ν*_*ofs*_ *MW*_*o*_*C*^*os*^ → *ν*_*tfs*_ *MW*_*t*_*C*^*ts*^	[12,48]
Tumor destruction	*ν*_*tds*_ *MW*_*t*_*C*^*ts*^ + *ν*_*cds*_ *MW*_*c*_*C*^*cs*^ → *ν*_*eds*_ *MW*_*e*_*C*^*es*^ + *ν*_*wds*_ *MW*_*w*_*C*^*ws*^	[35,41]
Necrotic formation	*ν*_*tns*_ *MW*_*t*_*C*^*ts*^ → *ν*_*nns*_ *MW*_*n*_*C*^*ns*^	[3,12]
Necrotic lysis	*ν*_*nls*_ *MW*_*n*_*C*^*ns*^ → *ν*_*els*_ *MW*_*e*_*C*^*es*^ + *ν*_*wls*_ *MW*_*w*_*C*^*ws*^	[33]

**Table 2:** Composition of three-phase systems

Entity	Index	Species
Wetting phase	*w*	{*g*, *o*, *c*, *w*, *z*}
Non-wetting phase	*n*	{*l*, *n*, *g*, *o*, *c*, *w*, *x*}
Solid phase	s	{*e*, *g*, *o*, *w*, *y*}
Fluid–fluid interface	*wn*	{—}
Wetting fluid–solid interface	*ws*	{—}
Non-wetting fluid–solid interface	*ns*	{—}
Common curve	*wns*	{—}

**Table 3:** Reactions for three-phase systems

Description	Reaction	References
Tumor formation	*ν*_*gfn*_ *MW*_*g*_*C*^*gn*^ + *ν*_*ofn*_ *MW*_*o*_*C*^*on*^ → *ν*_*tfn*_ *MW*_*t*_*C*^*tn*^	[48,49]
Tumor destruction	*ν*_*tdn*_ *MW*_*t*_*C*^*tn*^ +*ν*_*cdn*_ *MW*_*c*_*C*^*cn*^ → *ν*_*edn*_ *MW*_*e*_*C*^*en*^ + *ν*_*wdn*_ *MW*_*w*_*C*^*wn*^	[4,35]
Necrotic formation	*ν*_*tnn*_ *MW*_*t*_*C*^*tn*^ → *ν*_*nnn*_ *MW*_*n*_*C*^*nn*^	[13,61]
Necrotic lysis	*ν*_*nln*_ *MW*_*n*_*C*^*nn*^ → *ν*_*eln*_ *MW*_*e*_*C*^*en*^ + *ν*_*wln*_ *MW*_*w*_*C*^*wn*^	[33,61]

## List of symbols

*A*: Chemical affinity
**C**: Green’s deformation tensor
*C*: Molar species concentration
$\hat{c}$: Nonnegative solid compressibility coefficient
$\hat{D}$: Second-rank symmetric dispersion tensor
**d**: Rate of strain tensor, $d=\left[\nabla v+{\left(\nabla v\right)}^{\text{T}}\right]/2$
*E*: Internal energy density
$\bar{E}$: Partial mass energy
$E_{**}^{\bar{\bar{\alpha }}}$: Macroscale entity-based total energy conservation equation, Eq. (3)
*F*: Smooth differential functional form
*f*: General scalar function
**G**: Geometric orientation tensor, $G_{\alpha }=I-I_{\alpha }^{\prime }$ for $\alpha \in J_{\text{I}}$, $G_{wns}=I-I_{wns}^{\prime \prime }$
$\overset{\kappa \to \alpha }{G_{0}}$: Macroscale transfer rate of potential energy due to variability of mass exchange per volume
**g**: Body force per unit mass, gravity
$\bar{H}$: Partial mass enthalpy
*h*: Energy source density
$h_{0}^{\bar{\bar{\alpha }}}$: Macroscale energy source density for entity *α* due to body force and velocity fluctuations
**I**: Identity tensor
**I**′: Unit tensor in a surface, $I_{\alpha }^{\prime }=I-n_{\beta }n_{\beta }$ for $\alpha \in J_{\text{I}}$, $\beta \in J_{\text{c}\alpha }^{+}$
**I**″: Unit tensor in a common curve, $I_{wns}^{\prime \prime }=I_{wns}I_{wns}$
J: Set of entity indices
$J_{\text{c}\alpha }$: Connected set of indices for entity *α*, $J_{c\alpha }=J_{\text{c}\alpha }^{+}\cup J_{\text{c}\alpha }^{-}$
$J_{\text{c}\alpha }^{+}$: Connected set of indices of one dimension higher than entity *α*
$J_{\text{c}\alpha }^{-}$: Connected set of indices of one dimension lower than entity *α*
$J_{\text{f}}$: Set of fluid-phase indices
$J_{\text{I}}$: Set of interface indices
$J_{\text{P}}$: Set of phase indices
$J_{\text{rxn}i\alpha }$: Set of reactions involving the *i* species in the *α* entity
$J_{\text{rxn}\alpha }$: Set of reactions in the *α* entity
$J_{s}$: Set of species indices
$J_{s\backslash N}$: Set of species indices except species *N*
$J_{\backslash S}$: Set of entity indices except the solid phase
$J_{\alpha }^{\alpha \beta }$: Macroscale surface curvature, $J_{\alpha }^{\alpha \beta }={\langle \nabla^{\prime }\cdot n_{\alpha }\rangle }_{\Omega_{\alpha \beta },\Omega_{\alpha \beta }}$ for $\alpha ,\beta \in J_{\text{f}}$
*j*: Jacobian
$\hat{K}$: Nonnegative reaction rate coefficient
*K*: Gaussian curvature
*K*_*E*_: Kinetic energy per mass due to velocity fluctuations
${\hat{K}}_{\text{M}}$: Mass transfer coefficient
${\hat{k}}_{1}^{wn}$: Parameter for rate of relaxation of interfacial area
**l**: Unit vector tangent to a common curve
$\overset{i\kappa \to i\alpha }{M}$: Macroscale transfer rate of mass of species *i* in entity *κ* to species *i* in entity *α* per volume
$M_{**}^{\bar{\bar{i\alpha }}}$: Macroscale species mass conservation equation, Eq. (1)
*MW*: Molecular weight
*N*: Number of chemical species
**n**: Unit normal vector
$P_{**}^{\bar{\bar{\alpha }}}$: Macroscale entity-based momentum conservation equation, Eq. (2)
*p*: Pressure
$\overset{\kappa \to \alpha }{Q_{1}}$: Macroscale transfer rate of internal energy from entity *κ* to entity *α* per volume
$\overset{\kappa \to \alpha }{Q_{1}^{*}}$: Macroscale energy exchange between entities which dimensionalities differ by two
**q**: Non-advective energy flux
**q**_**g**0_: Non-advective energy flux associated with mechanical processes
*R*: Molar rate of reaction
$\hat{R}$: Positive definite resistance tensor
*R*_*g*_: Ideal gas constant
*r*: Mass production rate density
$\overset{\kappa \to \alpha }{T_{0}}$: Macroscale transfer rate of momentum from entity *κ* to entity *α* per volume
$\overset{\kappa \to \alpha }{T*}$: Macroscale momentum exchange between entities which dimensionalities differ by two
**t**: Stress tensor
*t*: Time
**u**: Diffusion/dispersion velocity, $u_{i\alpha }=v_{i\alpha }-v_{\alpha },u^{\bar{\bar{i\alpha }}}=v^{\bar{i\alpha }}-v^{\bar{\alpha }}$
**v**: Velocity
*W*: Weighting function for averaging
**w**: Velocity of a domain boundary
*x*: Mole fraction

## Greek letters

*γ*: Interfacial or common curve lineal tension
*ϵ*: Porosity
$\epsilon^{\bar{\bar{\alpha }}}$: Specific entity extent measure
*θ*: Temperature
*κ*_*G*_: Geodesic curvature, *κ*_*Gwns*_ = **l**_*wns*_ · ∇″**l**_*wns*_ · **n**_*ws*_
*κ*_*N*_: Normal curvature, *κ*_*Nwns*_ = **l**_*wns*_ · ∇″**l**_*wns*_ · **n**_*s*_
Ʌ: Entropy production rate
*μ*: Chemical potential
*ν*: Molar stoichiometric reaction coefficient
*ρ*: Mass density
$\varphi^{\bar{\bar{ws,wn}}}$: Macroscale measure of contact angle
$\chi_{s}^{\bar{\bar{\alpha s}}}$: Fraction of the solid surface in contact with fluid phase *α*, $\chi_{s}^{\bar{\bar{\alpha s}}}=\epsilon^{\bar{\bar{\alpha s}}}/\left(\epsilon^{\bar{\bar{ws}}}+\epsilon^{\bar{\bar{ns}}}\right)$, $\alpha \in J_{\text{f}}$
$\chi^{\bar{\bar{n}}}$: Specific Euler characteristic of the non-wetting fluid phase, defined in Eq. (57)
*ψ*: Body force potential per mass
Ω: Spatial domain
*ω*: Mass fraction

## Subscripts and superscripts

*c*: Chemotherapeutic drug species qualifier
*e*: Extra-cellular matrix species qualifier
*f*: Fluid phase qualifier
*f*_*s*_: Fluid–solid interface qualifier
*g*: Glucose species qualifier
*i*: Chemical species qualifier
*k*: Chemical reaction qualifier
*l*: Living tumor species qualifier
*n*: Non-wetting phase qualifier
*n*: Necrotic tumor species qualifier
*N*: Reference chemical species qualifier
*ns*: Qualifier for interface between non-wetting and solid phases
*o*: Oxygen species qualifier
*s*: Solid-phase qualifier
*w*: Wetting-phase qualifier
*wn*: Qualifier for interface between wetting and non-wetting phases
*wns*: Qualifier for common curve where wn, ws and ns interfaces meet
*ws*: Qualifier for interface between wetting and solid phases
*α*: Entity qualifier
*β*: Entity qualifier
*κ*: Entity qualifier
$\bar{\alpha }$: Superscript indicating mass average over entity *α*
$\bar{\bar{\alpha }}$: Superscript indicating uniquely defined average over entity *α*
∗: Indicates a concentrated force in an exchange between entities with a difference in dimensionality equal two
−: Above a superscript refers to a density weighted macroscale average
=: Above a superscript refers to a uniquely defined macroscale average
eq: Subscript indicating the property is at equilibrium
*w*: Water species qualifier
*x*: Collective background species qualifier for the non-wetting fluid phase
*y*: Collective background species qualifier for the solid phase
*z*: Collective background species qualifier for the wetting fluid phase

Subscripted entity/species qualifiers refer to microscale quantities, and superscripted entity/species qualifiers refer to macroscale quantities. If both entity/species subscript and superscript are present, the quantity is averaged over the entity boundary

## Other mathematical symbols

${\text{D}}^{\bar{\alpha }}/\text{D}t$: Material derivative of entity *α*, ${\text{D}}^{\bar{\alpha }}/\text{D}t=\partial /\partial t+v^{\bar{\alpha }}\cdot \nabla$
∇′: Microscale surficial del operator, $\nabla^{\prime }=I_{\alpha }^{\prime }\cdot \nabla$
∇″: Microscale curvilinear del operator, $\nabla^{\prime \prime }=I_{\alpha }^{\prime \prime }\cdot \nabla$
${\langle f_{\alpha }\rangle }_{\Omega_{\beta },\Omega_{\gamma }},W$: $=\underset{\Omega_{\beta }}{\int }Wf_{\alpha }\text{d}r/\underset{\Omega_{\gamma }}{\int }W\text{d}r$ General average of microscale property *f*_*α*_
${\langle f_{\alpha }\rangle }_{\Omega_{\beta },\Omega_{\gamma }}$: $={\langle f_{\alpha }\rangle }_{\Omega_{\beta },\Omega_{\gamma },1}$
$f_{\alpha }^{\beta }$: $={\langle f_{\alpha }\rangle }_{\Omega_{\beta },\Omega_{\beta }}$, General average
*f* ^*α*^: $={\langle f_{\alpha }\rangle }_{\Omega_{\alpha },\Omega_{\alpha }}$, Intrinsic average
$f_{\alpha }^{\bar{\beta }}$: $={\langle f_{\alpha }\rangle }_{\Omega_{\beta },\Omega_{\beta },\rho_{\alpha }}$, General density-weighted average
$f^{\bar{\alpha }}$: $={\langle f_{\alpha }\rangle }_{\Omega_{\alpha },\Omega_{\alpha },\rho_{\alpha }}$, Intrinsic density-weighted average
$f^{i\;\bar{\alpha }}$: $={\langle f_{i\alpha }\rangle }_{\Omega_{\alpha },\Omega_{\alpha },\rho_{\alpha }}$, Intrinsic density-weighted average
$f_{i\alpha }^{\bar{\beta }}$: $={\langle f_{i\alpha }\rangle }_{\Omega_{\beta },\Omega_{\beta },\rho_{\alpha }\omega_{i\alpha }}$, General species mass density weighted average
$f^{\bar{i\alpha }}$: $={\langle f_{i\alpha }\rangle }_{\Omega_{\alpha },\Omega_{\alpha },\rho_{\alpha }\omega_{i\alpha }}$, Intrinsic species mass density weighted average

## Abbreviations

SEI: Simplified entropy inequality
TCAT: Thermodynamically constrained averaging theory
